# Temporal Stability of Seagrass Extent, Leaf Area, and Carbon Storage in St. Joseph Bay, Florida: a Semi-automated Remote Sensing Analysis

**DOI:** 10.1007/s12237-022-01050-4

**Published:** 2022-11-01

**Authors:** Marie Cindy Lebrasse, Blake A. Schaeffer, Megan M. Coffer, Peter J. Whitman, Richard C. Zimmerman, Victoria J. Hill, Kazi A. Islam, Jiang Li, Christopher L. Osburn

**Affiliations:** 1 ORISE Fellow, Office of Research and Development, U.S. Environmental Protection Agency, Durham, NC, USA; 2 Department of Marine, Earth and Atmospheric Sciences, North Carolina State University, Raleigh, NC, USA; 3 Office of Research and Development, U.S. Environmental Protection Agency, Durham, NC, USA; 4 Department of Ocean and Earth Sciences, Old Dominion University, Norfolk, VA, USA; 5 Department of Electrical and Computer Engineering, Old Dominion University, Norfolk, VA, USA

**Keywords:** Seagrass, Submerged aquatic vegetation, Leaf area index, Carbon, Landsat, Trend analysis

## Abstract

Seagrasses are globally recognized for their contribution to blue carbon sequestration. However, accurate quantification of their carbon storage capacity remains uncertain due, in part, to an incomplete inventory of global seagrass extent and assessment of its temporal variability. Furthermore, seagrasses are undergoing significant decline globally, which highlights the urgent need to develop change detection techniques applicable to both the scale of loss and the spatial complexity of coastal environments. This study applied a deep learning algorithmto a 30-year time series of Landsat 5 through 8 imagery to quantify seagrass extent, leaf area index (LAI), and belowground organic carbon (BGC) in St. Joseph Bay, Florida, between 1990 and 2020. Consistent with previous field-based observations regarding stability of seagrass extent throughout St. Joseph Bay, there was no temporal trend in seagrass extent (23 ± 3 km^2^, *τ* = 0.09, *p* = 0.59, *n* = 31), LAI (1.6 ± 0.2, *τ* = −0.13, *p* = 0.42, *n* = 31), or BGC (165 ± 19 g C m^−2^, *τ* = − 0.01, *p* = 0.1, *n* = 31) over the 30-year study period. There were, however, six brief declines in seagrass extent between the years 2004 and 2019 following tropical cyclones, from which seagrasses recovered rapidly. Fine-scale interannual variability in seagrass extent, LAI, and BGC was unrelated to sea surface temperature or to climate variability associated with the El Niño-Southern Oscillation or the North Atlantic Oscillation. Although our temporal assessment showed that seagrass and its belowground carbon were stable in St. Joseph Bay from 1990 to 2020, forecasts suggest that environmental and climate pressures are ongoing, which highlights the importance of the method and time series presented here as a valuable tool to quantify decadal-scale variability in seagrass dynamics. Perhaps more importantly, our results can serve as a baseline against which we can monitor future change in seagrass communities and their blue carbon.

## Introduction

Seagrass meadows are considered important shallow marine ecosystems, reducing wave action, stabilizing sediments, and regulating nutrient loading (Beck et al. 2018). They also provide an important source of primary production for coastal food webs including microbes, dugongs, turtles, crustaceans, and snails and serve as habitats for myriads of fish and invertebrates. Although currently estimated to occupy less than 0.2% of the world’s oceans (Fourqurean et al. 2012), seagrass meadows are thought to sequester ~ 15% of all organic carbon buried in the sea (Kennedy and Björk 2009). Despite growing recognition of their carbon sink capacity (Oreska et al. 2017; Duarte and Krause-Jensen 2017; Lima et al. 2019; Prentice et al. 2019; Berger et al. 2020), seagrasses still represent the largest source of uncertainty in the global blue carbon stocks and inventories (Chmura et al. 2016) due to unreliable measures of worldwide seagrass area which span a 30-fold range from 150,000 to 4,320,000 km^2^ (Duarte 2017).

In addition to critical ecosystemservices, Florida seagrass meadows provide important economic services through recreational fishing, scalloping and tourismworth more than $20 billion per year (Orth et al. 2006). Approximately 10,000 km^2^ of seagrasses have been mapped in estuarine and nearshore Florida waters primarily in southern Florida (~ 6500 km^2^) and the Big Bend area (~ 2500 km^2^). The western Florida Panhandle, which includes St. Joseph Bay, supports an estimated 160 km^2^ of seagrasses, representing 2% of the total Florida seagrass inventory (Yarbro and Carlson 2016). Global seagrass populations have been estimated to be declining at a rate of 7% year^−1^ since 1990 largely due to sustained pressure from coastal development, human activities, and declining water quality (Waycott et al. 2009; Duarte et al. 2018; Evans et al. 2018). Survey data from St. Joseph Bay indicate that, in contrast to the global trend, seagrass extent has been stable primarily because urbanization and eutrophication in the area have been relatively minor (Yarbro and Carlson 2016).

Even in the absence of local anthropogenic disturbance, changing climate forces may threaten the overall health and stability of St. Joseph Bay’s seagrass meadows and their carbon storage, prompting the need for frequent assessment of their areal coverage, density, and biomass. St. Joseph Bay’s location in the northern Gulf of Mexico makes it vulnerable to potential climate impacts such as ocean warming (Wanninkhof et al. 2015; Laurent et al. 2018; Feely et al. 2018), which may be moderated by ocean acidification (Zimmerman 2021). However, the infrequent temporal coverage of past surveys has precluded an examination of the impact of larger scale drivers of climate variability, such as the El Niño-Southern Oscillation (ENSO) and the North Atlantic Oscillation (NAO), on seagrass areal coverage and density. While the elevated water temperatures associated with ENSO events have been shown to drive seagrass mortality in some studies (Seddon et al. 2000; Carlson et al. 2018), others have instead reported a corresponding rise in seagrass biomass and productivity (Nelson 1997; Lin et al. 2018). Although the thermal stress associated with such events seems to be the main threat to seagrasses in locations with restricted water circulation (Koch et al. 2007; Hall et al. 2016) such as St. Joseph Bay, a cold-water event associated with a change in the NAO was correlated with large-scale seagrass decline in Bermuda (Murdoch et al. 2007).

Mapping seagrass distributions from in situ plots, transect measurements, or photointerpretation can be extremely labor-intensive. Further, they often lack the temporal and spatial coverage required to assess large-scale seagrass dynamics across time and space. Retrospective seagrass mapping through satellite remote sensing can yield more accurate assessments of trends in growth and decline (Veettil et al. 2020) across the whole system by providing a regular time series of spatial observations from which seagrass cover and biomass can be determined (Baumstark et al. 2013). Changes in seagrass cover have been assessed using various high spatial resolution satellite platforms, including Planet Labs’ RapidEye satellites (Traganos et al. 2018) and DigitalGlobe’s (now Maxar) WorldView-2 (Coffer et al. 2020), IKONOS and Quickbird-2 satellites (Kovacs et al. 2018), but the temporal range of these platforms is usually limited. The Landsat series, on the other hand, is the most widely used satellite platform for seagrass applications due to its extensive historical archive (1972 to present), providing the ability to analyze long-term trends in seagrass spatial extent (Ferguson and Korfmacher 1997; Wabnitz et al. 2008; Lyons et al. 2011; Roelfsema et al. 2014; Hossain et al. 2015; Misbari and Hashim2016; Kovacs et al. 2018; Borfecchia et al. 2019; Ha et al. 2021). In addition to classifying seagrass cover across the landscape, the multispectral nature of Landsat imagery allows retrieval of seagrass leaf area index (LAI) on a per-pixel basis using the brightness in the green band (band 2 in Landsat 5–7, band 3 in Landsat 8), enabling the quantification of seagrass carbon stocks, in addition to areal extent, across the submarine landscape (Dierssen et al. 2003; 2010; Bresciani et al. 2011; Hill et al. 2014). LAI is an important indicator of potential photosynthetic activity and is strongly correlated to shoot density and standing biomass.

Although remote sensing of seagrass extent is well documented, studies on seagrass blue carbon stock mapping and estimations using satellite-based remote sensing are limited. To explicitly address the role that blue carbon ecosystems such as seagrasses play in climate change mitigation, their blue carbon inventories must be adequately quantified. With the current need for more effective mapping of blue carbon, a wide range of satellite-based remote sensing approaches have been developed using the correlation between biomass or LAI and spectral responses and carbon conversion factors (Wicaksono and Hafizt 2013; Hill et al. 2014; Tamondong et al. 2018). Other studies have demonstrated the use of multispectral imagery with deep learning methods to map seagrass carbon stocks (Hossain et al. 2015; Misbari and Hashim2016; Tamondong et al. 2018; Phamet al. 2019; Ha et al. 2021; Stankovic et al. 2021).

Although several studies have used Landsat imagery to map temporal changes in the extent of seagrass in different parts of the world over time periods ranging from25 to 40 years (Dekker et al. 2005; Gullström et al. 2006; Lyons et al. 2012; Roelfsema et al. 2014; Leblanc et al. 2021; Ha et al. 2021), few of them have used continuous images over their study period. The temporal gaps and labor-intensive analyses involved in these studies highlight the need for greater temporal resolution in tandem with efficient semi-automated seagrass detection techniques. Unlike manual analyses, deep learning algorithms (Bui et al. 2020) now appear to provide a pathway for semi-automated classification of seagrass presence within individual image pixels (Islam et al. 2020; Moniruzzaman et al. 2019; Coffer et al. 2020) which can provide consistent, reproducible, and expeditious measurements of seagrass extent, meadow shape, and patch connectivity (Pham et al. 2017; Ha et al. 2020, 2021).

To fill the current mapping and monitoring gaps in both seagrass extent and blue carbon inventories, the purpose of this study was to quantify the temporal variation in seagrass extent, LAI and belowground organic carbon (BGC) in St. Joseph Bay, Florida (FL), USA, using a temporally consistent (1 image per year) 30-year time series of Landsat imagery and explore the potential impacts of ENSO and NAO, sea surface temperature (SST) and tropical cyclones on seagrasses and their carbon storage. Extensive in situ optical studies previously conducted in St. Joseph Bay (Hill et al. 2014; Schaeffer et al. 2015; Conmy et al. 2017) and the potential for increased herbivory by green sea turtles to limit seagrass abundance in the future (Rodriguez and Heck 2020) make St. Joseph Bay an ideal area of interest for exploring the ability of Landsat satellite imagery to quantify temporal patterns of seagrass extent in coastal ecosystems. The specific objectives of this study were to (i) map seagrass extent using a machine learning classification method; (ii) quantify temporal change or stability in the satellite-estimated seagrass extent, LAI and BGC at a local scale between 1990 and 2020; and (iii) to explore the potential long-term impacts of ENSO, NAO, SST, and tropical cyclones on the seagrass population in St. Joseph Bay. The 30-year time period considered in this study is sufficient for generating climate-relevant time series, typically referred to as climatic time series.

## Methods

A list of abbreviation used throughout this study is provided in Table S1 (supplemental information).

### Study Site

**Fig. 1:**
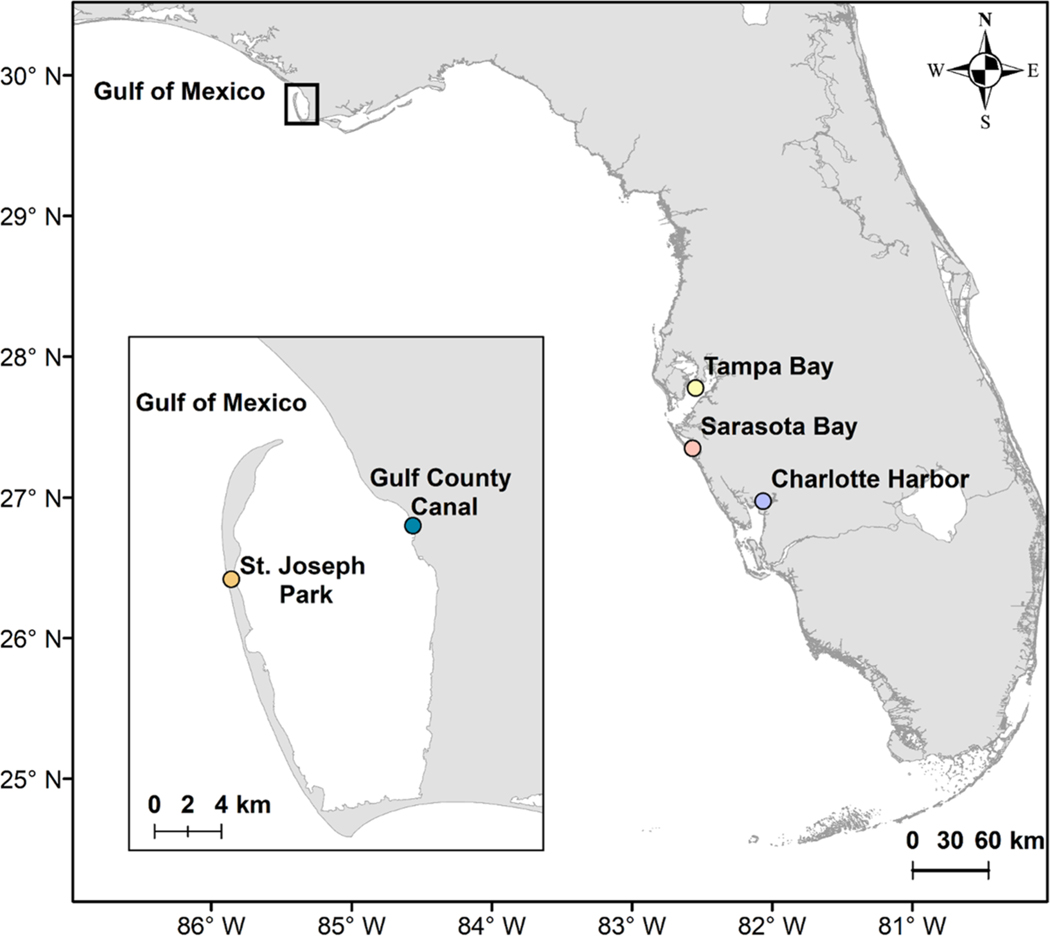
Map of the Florida Gulf Coast (USA) with St. Joseph Bay located inside the red rectangle. Inset shows St. Joseph Bay as our study site for this research and the location of St. Joseph Park where Major Hurricane Michael in October 2018 generated waves and currents strong enough to bisect the peninsula adjacent to the park, creating a large passage from St. Joseph Bay into the Gulf of Mexico. Inset also shows the location of the Gulf County Canal where suspended sediments and chromophoric dissolved organic matter (CDOM) create an optically challenging environment for remote sensing analysis of St. Joseph Bay waters. The locations of Sarasota Bay, Tampa Bay and Charlotte Harbor, where long-term runoff-related impacts from hurricane events caused pronounced declines in seagrass abundance (Carlson et al. 2010) are also shown

St. Joseph Bay is a shallow, subtropical lagoon covering ~ 200 km^2^ on the northwestern Florida panhandle (29.8° N, 85.5° W). It is bounded on the west by the St. Joseph Peninsula, on the east by the Florida mainland and opens north to the Gulf of Mexico (Fig. 1). Salinities in the Bay range from 22 to 35 while water temperatures range from winter lows around 8.5 °C to summer highs around 32 °C (Bologna 1998). St. Joseph Bay has a mean depth of 6.4 m, with a maximum depth of 10.7 m near the northern tip of the Peninsula. The southern part of the Bay provides a broad, shallow habitat that contains extensive seagrass meadows interspersed with patches of bare sand at an average depth of 0.9 m(Stewart and Gorsline 1962; Valentine and Heck 1993). At least four seagrass species are present in the coastal waters of West Florida, namely turtlegrass (*Thalassia testudinum*), shoal grass (*Halodule wrightii*), manatee grass (*Syringodium filiforme*), and paddle grass (*Halophila* spp.) (Iverson and Bittaker 1986). Turtlegrass is the dominant species in St. Joseph Bay, followed by shoal grass and manatee grass (Savastano et al. 1984; Yarbro and Carlson 2016). Paddle grass has not been observed in St. Joseph Bay (Zimmerman and Hill, pers. obs.). The waters of St. Joseph Bay have been heavily influenced by suspended sediment and chromophoric dissolved organic matter (CDOM) since the construction of the Gulf County Canal (Fig. 1 inset) in 1938 (Stewart and Gorsline 1962), which may create an optically challenging environment for remote sensing analysis (Conmy et al. 2017).

### Satellite Image Processing

A total of 31 Landsat images were used in this analysis, including 22 Landsat 5 (L5) Thematic Mapper (TM) scenes spanning 1990 to 2011, one Landsat 7 (L7) Enhanced Thematic Mapper (ETM+) scene from 2012, and eight Landsat 8 (L8) Operational Land Imager (OLI) scenes spanning 2013 to 2020 (Table S2, supplemental information). Priority was given to images that were collected with less than 10% cloud cover during the meteorological autumn (September to November) to minimize confounding the long-term temporal trend with any inherent seasonal variations. However, a June image was used for 1998, an August image for 2007, a June image for 2009, and a March image for 2018 and 2020 because cloud-free autumn scenes were not available (Table S2, supplemental information). While sun glint on water surfaces is common in nadir-viewing satellites like Landsat 5–8 (Vanhellemont 2019), the sun-sensor-target angles of the Landsat images used in this study produced no apparent sun glint within our area of interest.

**Fig. 2:**
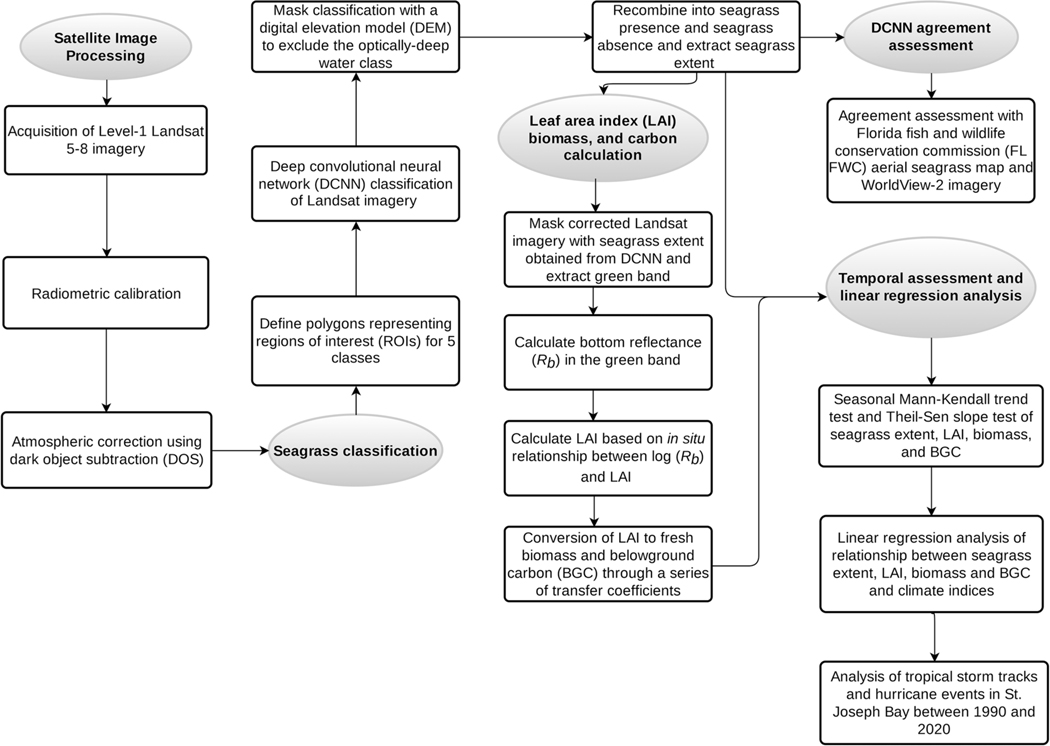
Flowchart of methods used in the present study to process and classify Landsat satellite data into seagrass extent and to compute leaf area index (LAI), biomass, and belowground organic carbon (BGC)

For each scene, collection-1 Level-1 Terrain and Precision data (L1TP) were obtained from the United States Geological Survey (USGS) Earth Explorer website (earthexplorer.usgs.gov). Spectral bands 1 through 5 and 7 were retained from the TM and ETM+ images and spectral bands 1 through 7 were retained from the OLI images, each with a spatial resolution of 30 m. All scenes were cropped to the extent of St. Joseph Bay (Fig. 1 inset). Spatial gaps in the L7 image, resulting from the scanline correction failure in May 2003, were interpolated using the ‘Fix Landsat 7 Scanline Errors’ tool provided by the ArcGIS Landsat Toolbox (Daniels 2012). Atmospheric correction was performed via dark object subtraction (DOS; Chavez 1988) following the semi-automated workflow described in Coffer et al. (2020), but instead using the near-infrared (NIR) band to retrieve the dark pixel value, as recommended in previous studies (e.g., Green et al. 2000). The use of the NIR band ensured that both land and inland water bodies were masked out to prevent the dark pixel value from being selected from inland waters. All satellite data processing and visualization were done in IDL 8.7 and ENVI 5.5 (Exelis Visual Information Solutions, Boulder, Colorado). The RStoolbox package (Leutner et al. 2017) in RStudio (R Core Team2017) was used to extract the spectral values from the corrected imagery for analysis. The image processing steps described above are summarized in Fig. 2 and the supplemental information (Supplemental information text S1 and S2).

### Seagrass Classification

A deep convolutional neural network (DCNN), previously detailed in Islamet al. (2020), was used to sort all pixels in the image to the following five classes: (i) land, (ii) optically-deep water that contained no bottom reflectance signatures, (iii) seagrass, (iv) submerged sand, and (v) intertidal. The intertidal class represented the transition areas between land and water that varied among images depending on the tidal stage at the time of acquisition. A threefold cross-validation task, published in Islamet al. (2020), showed that the DCNN achieved a superb performance, with 99% accuracy for St. Joseph Bay, justifying the use of the DCNN for our temporal seagrass analysis. Details of the structure of the DCNN and results of a sensitivity analysis used to select its input parameters can be found in the supplemental information (Supplemental information text S3 and S4).

**Fig. 3:**
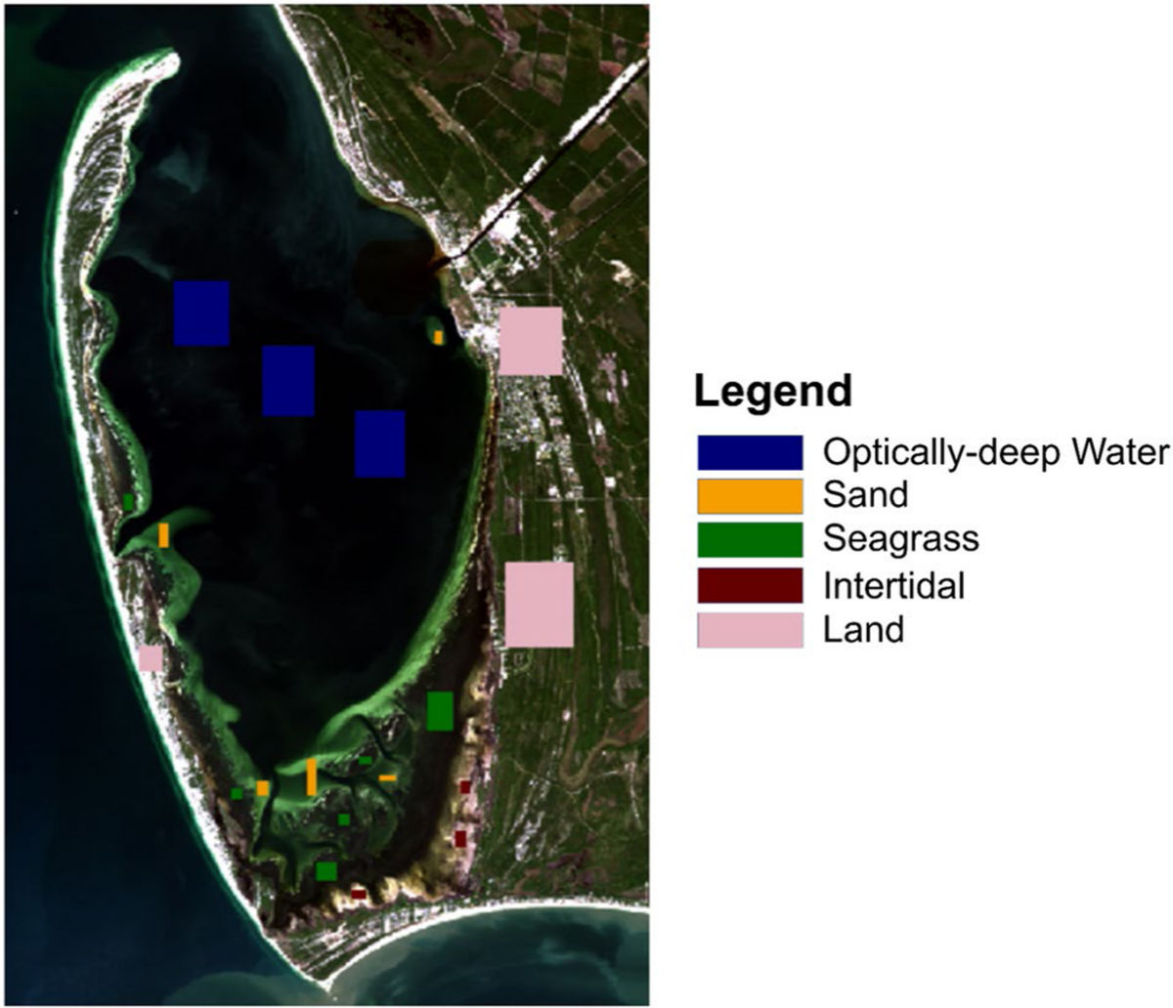
Regions of interest (ROIs) for each of the five classes overlaid on a Landsat 8 image from 26 October 2013. The same ROI locations were used across all images analyzed in this study

Known training classes were provided to the DCNN through spectral information contained in homogeneous regions of interest (ROI), which were defined through a combination of local expert knowledge (Zimmerman and Hill, pers. obs.), expected spectral characteristics (Figs. S1 and S2, supplemental information) and georeferenced in situ counts (Hill et al. 2014). Three to six ROIs were used for each of the five classes in each image (Fig. 3). The number of pixels within each ROI is listed in Table 1. Since the number of pixels within each ROI is imbalanced, the DCNN balances the input classes by randomly upsampling the data in each ROI to 20,000 pixels to achieve the same amount of spectral information among classes. Ninety percent of the data within these ROIs were used to train the DCNN model and the remaining ten percent were used for testing, as commonly employed in deep learning methods (Acharya et al. 2017). The same ROI locations were used to separately train the model for each scene to avoid producing differences in classification results between sensors or scenes because of differences in training ROI locations. Visual review confirmed that the locations of ROIs matched their intended class in each scene of the time series.

**Fig. 4:**
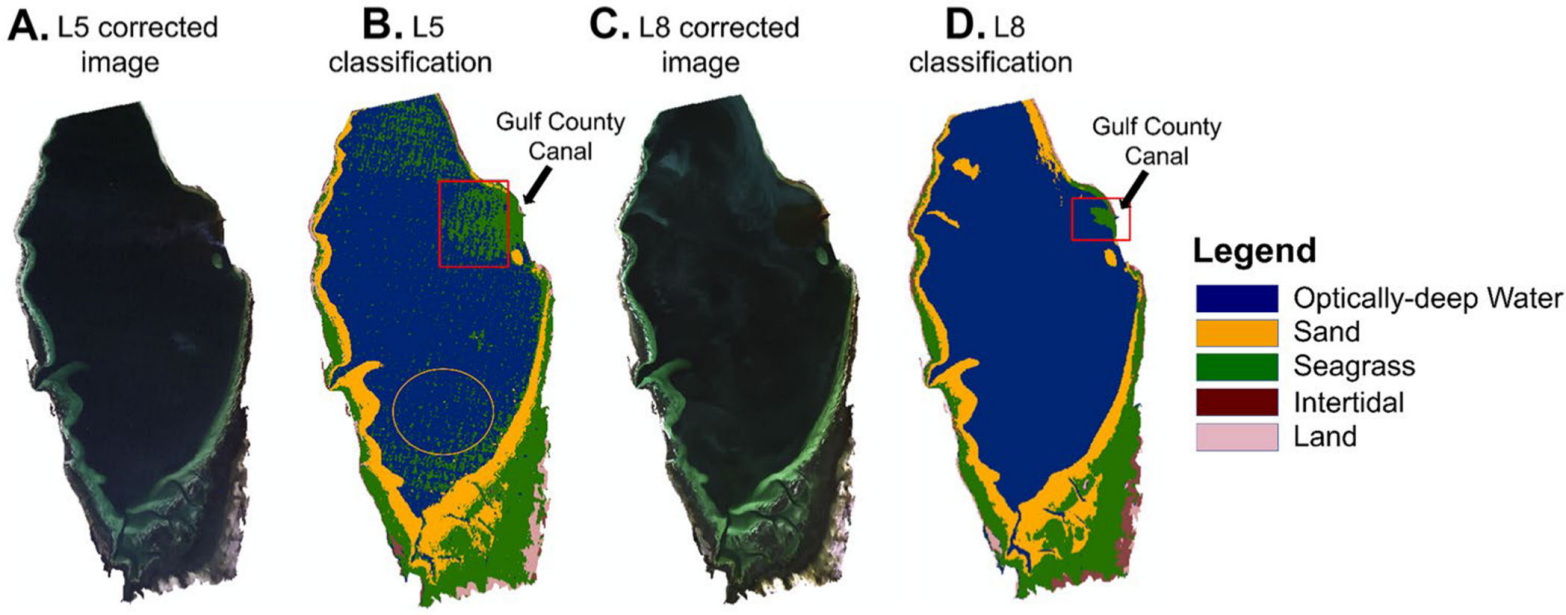
Example true color images and resulting classification maps of St. Joseph Bay derived from Landsat 5 (L5) and Landsat 8 (L8), including (**A**) a L5 true color image from 14 October 1991, (**B**) the resulting classification map from the L5 image where the black arrow indicates the location of the Gulf County Canal, (**C**) a L8 true color image from 26 October 2013, and (**D**) the resulting classification map from the L8 image. Red boxes indicate examples of pixels misclassified as seagrass in the optically-deep water, especially around the Gulf County Canal. The yellow circle indicates an area where optically-deep water pixels were misclassified as seagrass in L5 due to banding issues

The DCNN was not always able to distinguish seagrass pixels from optically-deep water pixels with high CDOM concentration, primarily those in the area surrounding the Gulf County Canal (Fig. 4). Therefore, to be consistent across sensors, we excluded the optically-deep water class from all Landsat images using a bathymetric digital elevation model (DEM) (Hill et al. 2014) of the Bay that masked all pixels at depths > 2.5 m from the analysis. The DEM raster was constructed in ArcGIS by interpolating data downloaded from the National Oceanic and Atmospheric Administration’s (NOAA) bathymetric data viewer (https://maps.ngdc.noaa.gov/viewers/bathymetry/) and the National Oceanographic Survey Hydrographic Survey H09924 (1981), H09925 (1981), and H09989 (1982).

Seagrass area coverage (km^2^) was computed for each Landsat image as the number of resolvable seagrass pixels multiplied by the area of each 30-mpixel (900 m^2^). The relationship between the tidal height at the time of image acquisition and seagrass surface area was investigated to determine if tidal stage had an influence on the seagrass area detected by the DCNN. Tidal heights were obtained from the NOAA tides and currents database (Port St. Joe, Station 8728912, www.tidesandcurrents.noaa.gov).

Feature importance was also assessed to determine the contribution of each Landsat band to the DCNN classification using the Shapley additive explanation values (SHAP) described in Lundberg and Lee (2017) and Padarian et al. (2020). A SHAP value is the average marginal contribution of each Landsat band to the DCNN model predictions among all possible combinations of Landsat bands. The SHAP method iterates through all combinations of Landsat bands to determine the impact of each combination on the resulting DCNN classification. These feature importance results were presented as percent importance of each band (Fig. S3, supplemental information).

### DCNN Agreement Assessment

Performance of the DCNN algorithm was assessed for L5 and L8. While photointerpreted aerial imagery was desired for comparison, there was only one available seagrass map for St. Joseph Bay spanning 1990–2020. Therefore, high spatial resolution satellite imagery was also used for comparison because the goal was to validate a classification from each sensor due to possible differences between Landsat sensors. Cross-satellite validation is a commonly accepted approach and has been successfully demonstrated in Duan et al. (2017) and Page et al. (2018). The lack of coincident photointerpreted aerial imagery and high spatial resolution satellite imagery precluded any performance assessment for L7. The DCNN classification of a L5 image collected at low tide on 19 Nov 2010 was compared to a seagrass map generated by the Florida Fish and Wildlife Conservation Commission (FL FWC). The 2010 FL FWC seagrass map was generated from a red–green–blue color aerial photographic image acquired at high tide in October 2010 by the Florida Department of Transportation (Statistics Canada 2008; Great Britain 2009). Visible features in the photographic image were interpretatively mapped by Quantum Spatial (2010, formerly Photo Science Inc.). The 2010 FL FWC aerial seagrass map was obtained as a shapefile (geodata.myfwc.com) and rasterized to match the 30-mspatial resolution of L5. The FWC seagrass map contained polygons defining both continuous and patchy seagrass intermixed with bare sand, which were all combined into a single class representing seagrass. The remaining pixels were reclassified as no seagrass. Nearly coincident scenes from L5 (19 Nov 2010) and Maxar’s WorldView-2 (WV2) satellite (14 Nov 2010) and from L8 (26 October 2013) and WV2 (24 October 2013) were also used for a cross-sensor validation of classified seagrass presence and absence between Landsat imagery and higher spatial resolution satellite imagery.

Agreements between the 2010 L5, 2010 FL FWC survey and 2010 WV2 classified images as well as between the 2013 L8 classified image and its coincident 2013 WV2 classified image were determined using the overall accuracy, the *K*(kappa) coefficient, precision, recall, and *F*-measure (Hossin and Sulaiman 2015). These metrics have been commonly used in evaluating machine learning classification performance (Picek et al. 2019). Overall agreement represents the number of pixels labeled as the same class between datasets, normalized to the total number of pixels in the scene. *K* is a measure of how well the DCNN classification performed compared to a random classification (Goodman and Kruskal 1954; Cohen 1960). It ranges between − 1 for perfect disagreement between datasets and 1 for perfect agreement with zero representing the performance of a random classification. Precision represents the fraction of DCNN-classified seagrass pixels that were also classified as seagrass in the aerial photointerpretation or WV2 image. Recall quantifies the fraction of all the labeled seagrass detected by the DCNN and the *F*-measure combines precision and recall into a single score that seeks to balance the concerns of those two metrics as the harmonic mean (reciprocal of the arithmetic mean). The *F*-measure ranges from 0 to 1 and the values are assessed like those for precision with 1 indicating a perfect agreement. Additionally, the non-parametric McNemar test (McNemar 1947) was used to statistically compare the agreement assessment between the 2010 L5-FWC seagrass classifications and the 2010 L5-WV2 seagrass classifications.

### Leaf Area Index, Fresh Biomass, and Carbon Calculation

Although spectral indices and soil radiometric indices have commonly been employed in retrieving LAI and aboveground carbon in intertidal seagrasses (Xue and Su 2017; Phamet al. 2020; Zoffoli et al. 2020; Ha et al. 2021), they are not appropriate for assessment of subtidal seagrasses because the water column dramatically attenuates the NIR reflectance, on which these indices rely, from the submerged plants and/or sediments. Hill et al. (2014) employed the Normalized Difference Vegetation index to map the narrow band of emergent vegetation at low tide, but the presence of an overlying water column prevented the use of this index to determine LAI across the aquatic landscape. For this reason, we chose a method that had been developed and successfully validated specifically for the subtidal seagrass populations in St. Joseph Bay using in situ LAI measurements and reflectance in Landsat’s green band (Hill et al. 2014). This method retrieves LAI for each seagrass pixel based on the previously developed relationship between bottom reflectance (log (*R*_b_)) and seagrass LAI (Dierssen et al. 2003), using georeferenced, in situ measurements from St. Joseph Bay, and other sites across the Florida Panhandle (Hill et al. 2014). The strong negative relationship between log (*R*_b_) and LAI (*r*^2^ = 0.81) provided the mathematical basis for LAI retrieval from the satellite imagery (Hill et al. 2014). Although the true units of LAI are m^2^ leaf per m^2^ seabed, LAI is usually reported as unitless and we will follow this convention throughout the manuscript. The calculation of *R*_b_ from remote sensing reflectance (*R*_rs_) in Landsat’s green band (560 nm) requires inputs of water depth from the NOAA DEM, the upwelling diffuse attenuation coefficient (*K*_Lu_), and the spectral diffuse water column attenuation coefficient (*K*_d_). *K*_d_ was calculated at 560 nm (to match the wavelength of Landsat’s green band) for the day of image acquisition from the *GrassLight* model (Zimmerman et al. 2015) based on chlorophyll *a* (Chl *a*, μgL^−1^), the CDOM absorption coefficient at 443 nm (m^−1^), and turbidity (NTU) values obtained from station SJB03 from the University of South Florida Virtual Buoy System (VBS, Hu et al. 2013). To account for changes in tidal height at time of image acquisition, the DEM was adjusted for tide height for each scene as the depth of the overlying water affects the retrieval of LAI, and the distorting effects of the overlying water column need to be removed to accurately quantify *R*_b_. *K*_Lu_ was calculated as *K*_d_/2π. The VBS data only went back to 2002. Therefore, we used the 2002–2020 data to determine the stability of *K*_d_ and *K*_Lu_ over time. Then, their averages were used to calculate *R*_b_ and therefore LAI for the years 1990 to 2001.

Seagrass carbon stocks were quantified from the LAI maps using a series of transfer coefficients that successively converted LAI at each seagrass pixel to fresh biomass (Eq. 1; van Tussenbroek 1998), dry biomass (Eq. 2; Sfriso and Ghetti 1998) and finally to organic carbon (Eq. 3; Hemminga and Duarte 2000; Howard et al. 2014). Previous studies indicate seagrass LAI can be retrieved from hyperspectral imagery with an uncertainty of 10 to 20% and an additional 10 to 20% uncertainty has been associated with conversions from LAI to biomass (Dierssen et al. 2003; Hill et al. 2014). Although the carbon stock of a seagrass meadow includes both living (seagrass aboveground and belowground biomass) and sediment (detrital) carbon pools, this analysis focused only on belowground seagrass biomass as it often dominates the total carbon stock in seagrass communities (Larkumet al. 2006) and the allochthonous organic carbon contribution can vary by an order of magnitude, with no consistent relation to plant morphology, or seagrass species (Lavery et al. 2013; Mazarrasa et al. 2018). In large and robust species like turtlegrass, the majority of the biomass is found belowground in the form of an extensive root and rhizome system (Zieman and Zieman 1989; Kaldy and Duntn 2000). Belowground biomass was therefore estimated based on the 1:3 ratio for aboveground and belowground biomass due to the dominance of the seagrass meadows by turtlegrass (Zieman 1975, 1982, 1989) and the sedimentary character of St. Joseph Bay (Eq. 4; Folger 1972). Belowground biomass may reach up to 88% of the total seagrass biomass (Collier et al. 2020), depending on the area and multiple biological and environmental factors (Ricart et al. 2020).


(1) $$Aboveground\;fresh\;biomass\;(Gg)=500\left(gm^{-2}\;leaf\;\right)\;\times LAI\left(m^{2}\;leaf\;m^{-2}\;seabed\;\right)\}\times 10^{9}$$

(2) $$Aboveground\;dry\;biomass\;(Gg)=\;Aboveground\;fresh\;biomass\;\times 0.2\times 10^{9}$$

(3) Totalorganiccarboncontent(GgC)=Aboveground dry biomass×0.34

(4) Belowgroundorganiccarbon(GgC)=Abovegrounddrybiomass×3×0.34

Remotely sensed LAI and BGC values retrieved from a November 2010 L5 image were validated against in situ LAI and LAI-derived BGC measures collected in St. Joseph Bay in November 2010. In situ shoot counts were collected by divers using 0.05 m^2^ quadrats (approximately 20 quadrats at each location) randomly located in direction (0 to 360°) and distance (0 to 20 m) from twenty-four stations located throughout St. Joseph Bay. Mean shoot leaf area (m^2^ shoot^−1^) was determined by measuring the length and width of all leaves on one shoot that was harvested at random from each quadrat (approximately 20 shoots per station). Mean LAI (and BGC) were calculated for each station as the product of mean shoot density and mean shoot leaf area. Twenty-four pixels within a 20-mradius of the in situ station location were extracted from the L5 LAI and BGC rasters and compared to the direct count estimates using an analysis of variance (ANOVA) statistical test.

### Temporal Assessment and Linear Regression Analysis

A non-parametric seasonal Mann–Kendall test (Kendall 1938; Mann 1945) and the associated seasonal Kendall slope estimator (Sen 1968; Hirsch et al. 1982; Theil 1992) were used to evaluate long-term trends in the time series of seagrass areal extent, LAI, and BGC from1990 to 2020. The Mann–Kendall test statistic, *tau* (*τ*), indicates the strength of the monotonic change, ranging between − 1 and 1. It was interpreted according to Cohen (1988) for correlation coefficients where an absolute value of *τ* ≥ 0.5 indicates a strong trend; 0.3 ≤ *τ* < 0.5 indicates a moderate trend and 0.1 ≤ *τ* < 0.3 indicates a weak trend. Following Weatherhead et al. (1998), a gamma (*γ*) statistic was computed to capture the number of years of observations needed for the trend in the data to overcome variability in the data. The *γ* statistic has been used for several environmental applications (Henson et al. 2010; Coffer and Hestir 2019; Urquhart et al. 2017; Coffer et al. 2021). Trends were analyzed using R statistical software (R Core Team2017) with the rkt package (Marchetto 2017).

Additionally, a seagrass frequency assessment was calculated from the time series of seagrass area (Johansson 2016; Sherwood et al. 2017; Clark et al. 2017). This frequency metric is computed on a pixel-by-pixel basis to quantify the temporal frequency of seagrass occurrence throughout the 30-year time series. For each Landsat satellite pixel, seagrass frequency is computed as the proportion of the 31 satellite scenes considered in which the DCNN indicated seagrass was present. This metric offers information regarding the stability of seagrass occurrence throughout St. Joseph Bay.

### Multiple Linear Regression Analysis

The impacts of ENSO, NAO, and SST on seagrass areal extent, LAI, and BGC were explored using a stepwise multiple linear regression (MLR). MLR is by far the most common tool to analyze such observational data (in this study, seagrass extent, LAI, BGC, ENSO, and NAO) because it allows researchers to assess the strength of the relationship between an outcome and several predictor variables as well as the importance of each of the predictors to the relationship. Various research papers have used MLR as a valid statistical approach in studying the relationship between seagrass metrics and predictor variables (Unsworth et al. 2007; Gullströmet al. 2008). ENSO and NAO climate indices were obtained from the National Weather Service Climate Prediction Center (cpc.ncep.noaa.gov), and mean SST (Schaeffer et al. 2018) was obtained for the Bay from the Analysis Ready Data surface temperature product derived from Landsat imagery (www.usgs.gov). The impact of tropical cyclones was also explored by retrieving the date and timing of tropical storm and hurricane tracks over St. Joseph Bay from the National Hurricane Center (www.nhc.noaa.gov) and matching them to the date and timing of Landsat image acquisition.

## Results

### Seagrass Classification and Change Assessment

**Fig. 5:**
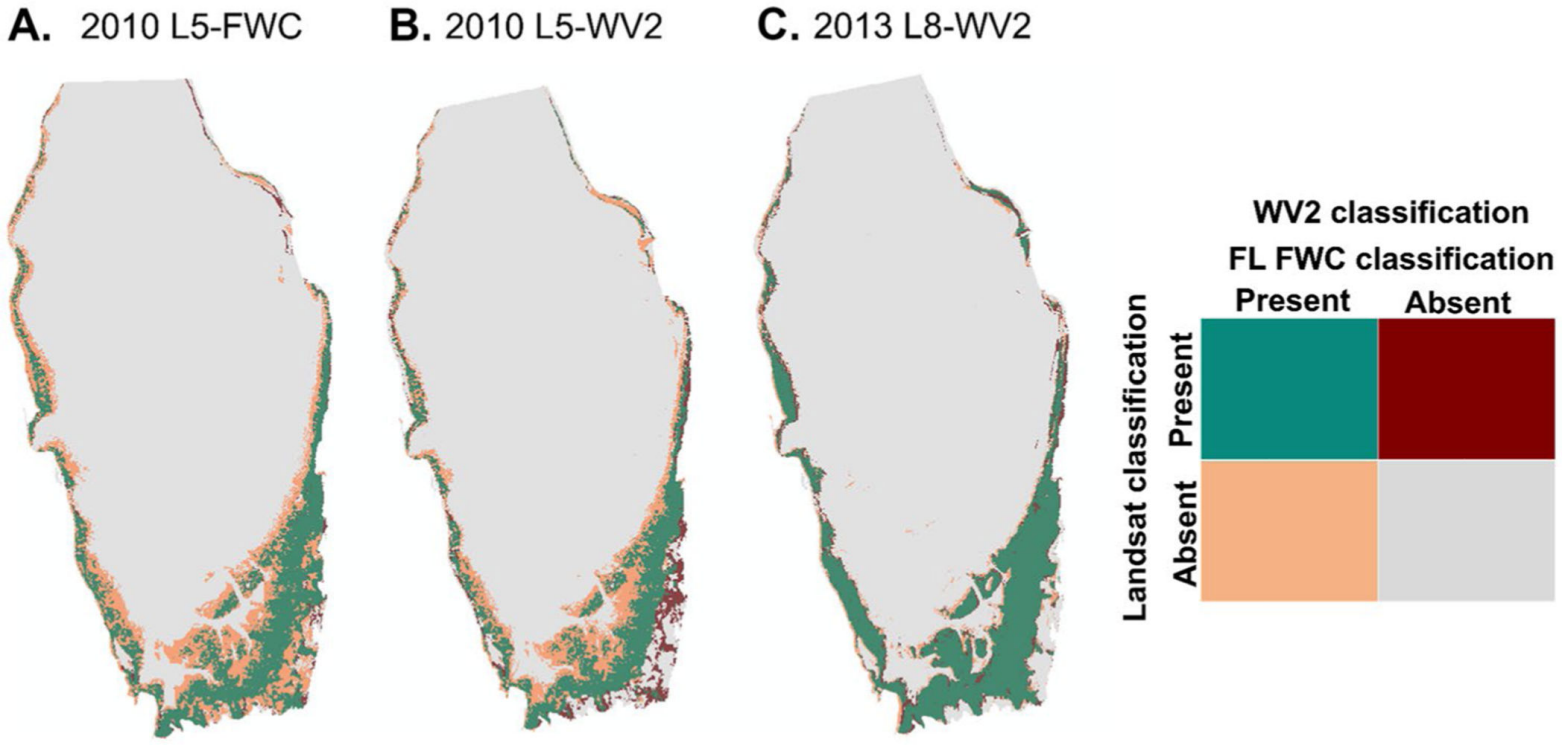
Difference maps showing areas of agreement in seagrass cover between (**A**) the 19 Nov 2010 Landsat 5 (L5) classification and the Oct 2010 Florida Fish and Wildlife Conservation Commission (FL FWC) classification, (**B**) the 19 Nov 2010 L5 classification and the 14 Nov 2010 WorldView-2 (WV2) classification, and (**C**) the 26 Oct 2013 Landsat 8 (L8) classification and the 24 Oct 2013 WV2 classification

Seagrass classification from L5 and L8 (Fig. 5) had a high degree of agreement with the 2010 FL FWC aerial survey and WV2 classification (Table 2). Comparison of seagrass extent derived from the 2010 L5 and 2010 FL FWC aerial image yielded precision and recall of 0.93 and 0.52 respectively for seagrass presence. The DCNN classification of seagrass in the 2013 L8 image yielded a precision, recall and *F*-measure of 0.88, 0.85 and 0.87 respectively, when compared to a 2013 WV2 image, also classified by the DCNN. Lower values of precision, recall, and *F*-measure of 0.77, 0.54, and 0.63, respectively, were obtained from the comparison of seagrass extent derived from the 2010 L5 image and the 2010 WV2 image, both classified by the DCNN. The McNemar test revealed a statistically significant difference (McNemar’s χ^2^ = 107.83, *p* < 0.0001) between the L5-FWC agreement assessment and the L5-WV2 agreement assessment. The contingency table (Table S3, supplemental information) showed that the L5-WV2 images had a stronger agreement than the L5-FWC images. In general, the *F*-measure for seagrass presence across all three validation datasets shows that the DCNN classification of L5 and L8 agreed with the validation datasets between 67 and 87% of the time (Table 2), and the DCNN algorithm was able to detect seagrass absence between 94 and 97% of the time.

### Seagrass Extent, LAI, Biomass and BGC Through Time

Values of Chl *a* and turbidity extracted from the VBS revealed no significant temporal trend. Further, the fact that their oscillations were 180° out-of-phase with each other (*r* = 0.5) tended to stabilize *K*_d_ and *K*_Lu_ over time (Fig. S4, supplemental information). *K*_Lu_ averaged 0.117 ± 0.01 and *K*_d_ averaged 0.734 ± 0.07 over the 18-year dataset. Although tidal height did not seem to influence the seagrass extent detected by the DCNN as evidenced by Fig. S5 (supplemental information), tidal height did vary by 85% over the 2002–2020 period (0.24 ± 0.21 m), potentially influencing LAI retrieval.

Although the DOS atmospheric correction performed well in separating the seagrass spectra from other classes for classification by the DCNN, the resulting *R*_rs_ values in the green band emanating from pixels classified as seagrass were brighter than those measured in situ by Hill et al. (2014). As a result, LAI, which is sensitive to the absolute magnitude of *R*_rs_, was underestimated compared to LAI measured in situ. To adjust for this difference in brightness, we derived the relationship between the LAI retrieved from a 14 Nov 2010 WV2 image (Hill et al., in prep) and a 19 Nov 2010 L5 image, using a linear regression analysis (Fig. S6, supplemental information). The slope between LAIs derived from the WV2 versus Landsat images was not different from 1, but the difference in brightness between the two scenes resulted in an offset of 0.98 that was subsequently used to correct the Landsat-derived LAIs.

Mean LAI derived from in situ observations and the L5 raster were identical (1.7), although the standard deviation for L5 raster data (σ = 0.1, *n* = 24) was an order of magnitude smaller than for the in situ counts (σ = 1.1, *n* = 72). Because BGC was derived from the LAIs, the ANOVA results showed that the mean BGC derived from in situ (172 g C m^−2^) and the L5 raster (179 g C m^−2^) data were statistically identical (F(1,23) = 0.65, *p* = 0.4).

**Fig. 6:**
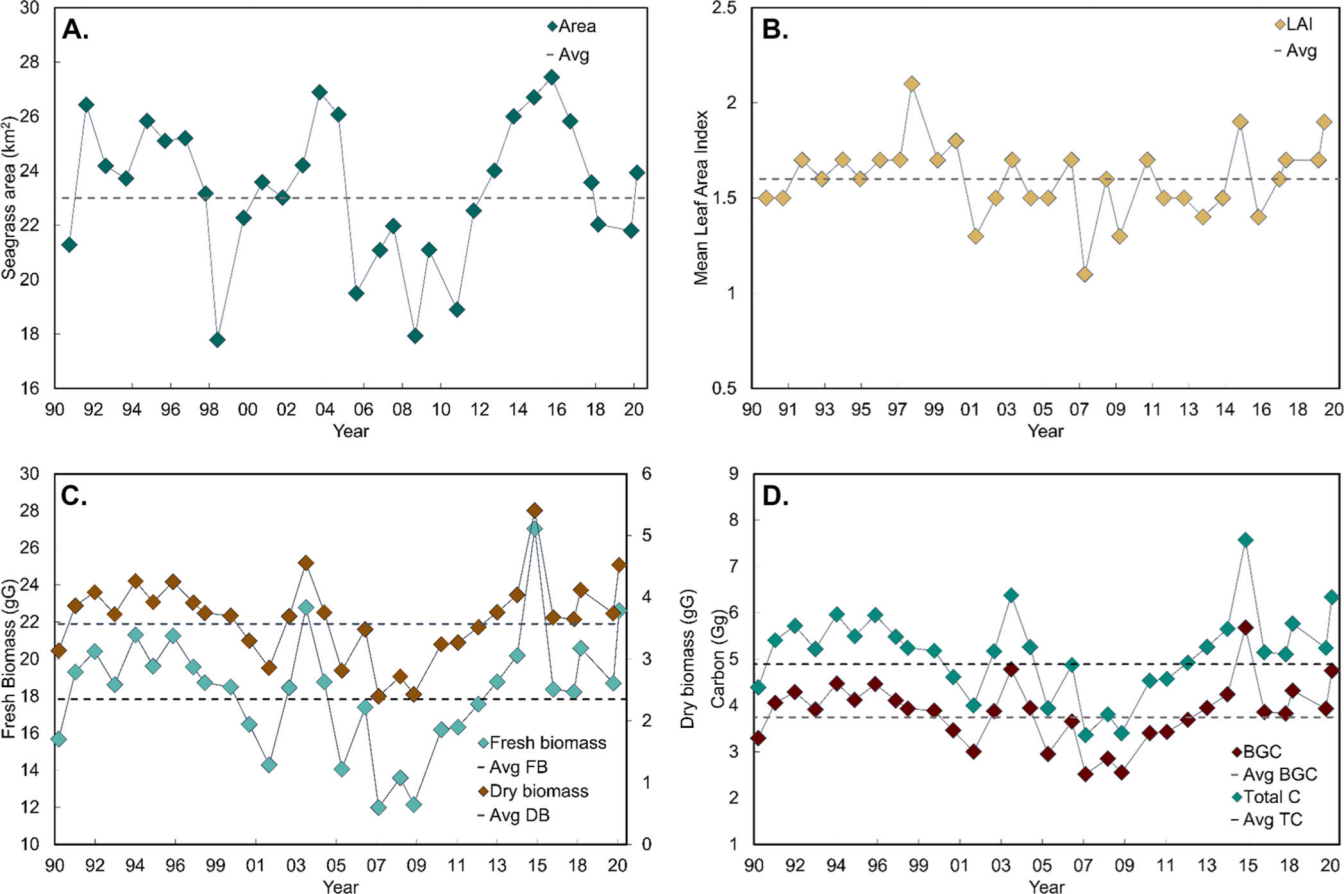
Time series of (**A**) seagrass area, (**B**) mean leaf area index (LAI), (**C**) fresh (FB) and dry biomass (DB), and (**D**) total organic carbon (TC) and belowground organic carbon (BGC) from 1990 to 2020 in St. Joseph Bay, FL. The dotted line represents the average for each parameter across the 30-year period

Our trend analysis revealed that seagrass extent in St. Joseph Bay remained stable over the 30-year period from 1990 to 2020 and covered an average of 23 ± 3 km^2^ of the Bay (Fig. 6a). The Theil-Sen test, used in conjunction with the seasonal Mann–Kendall test, revealed a slope value of 0.03 km^2^ year^−1^, which was not significantly different from 0 (*τ* = 0.09, *p* = 0.59, *n* = 31, Table 3). The *γ* statistic suggested that roughly 76 years of data would be needed for the current Theil-Sen slope to overcome variability in the data.

**Fig. 7:**
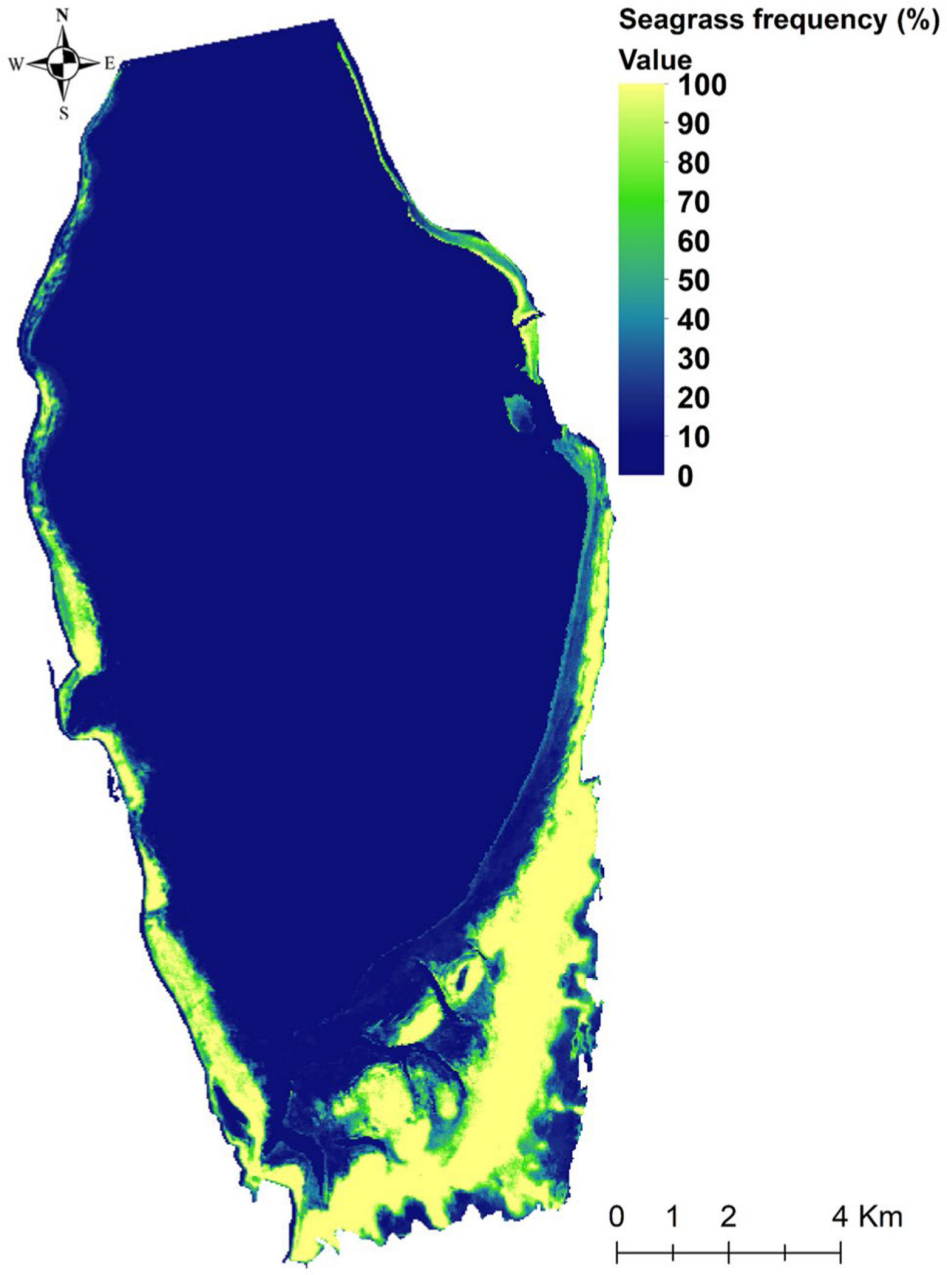
Temporal frequency assessment of seagrass changes between 1990 and 2020. A temporal frequency of 100% indicates satellite pixels at which seagrass was always present throughout the 30-year period when satellite imagery was acquired; a temporal frequency of 0% indicates satellite pixels at which seagrass was never detected throughout the 30-year period when satellite imagery was acquired. The color bar indicates where seagrass extent changed dynamically

Although there was no detectable long-term trend in seagrass abundance, visual inspection of the seagrass extent time series revealed three maxima and three minima over the 30-year time series, suggesting a decadal-scale periodicity. Seagrass covered an average of 23 ± 3 km^2^ of St. Joseph Bay during the first decade (1990–1999) of our analysis, with the lowest seagrass extent of 18 ± 0.3 km^2^ occurring in 1998. Seagrass extent expanded through 2004, reaching an area of 26 km^2^, then decreased to a low of 19 km^2^ in 2005, followed by a slow recovery that peaked around 2015 before declining into 2020 (Fig. 6a). A temporal frequency assessment revealed that most changes in seagrass extent occurred (i) in the intertidal transition region between land and fully submerged seagrass and (ii) along the deep edge of seagrass distribution (1.5 to 2 m depth; Fig. 7). Seagrass was present in the broad shallow areas between the intertidal and the deep edge almost 100% of the time.

Mean seagrass LAI retrieved from Landsat oscillated during the first two decades (1990–2010), with one maximum in 1998 above 2.0 and three minima in 2001, 2007, and 2009 around 1.2 (Fig. 6b). During the third decade (2010–2020), two maxima were observed in 2015 and 2020 and two minima were observed in 2013 and 2016, but these values were still within the range observed for the previous two decades (Fig. 6b). Overall, mean LAI ranged from 1.1 ± 0.2 to 2.1 ± 0.3, with an average of 1.6 ± 0.2. Although LAI and seagrass extent appeared to exhibit an inverse relationship in some years (e.g., 2012 to 2016, Fig. 6a, b), the overall variation in LAI was not correlated to seagrass extent (*r* = 0.1, *n* = 31, *p* = 0.60).

As with seagrass extent, we found no long-term trend in seagrass LAI over time (Table 3). The Theil-Sen test returned a slope value of 0, indicating no change. Because the *γ* statistic utilizes the Theil-Sen slope in its denominator, a slope of 0 corresponded to a *γ* statistic that also indicated no observable trend in LAI over the 30-year period (Table 3).

Total fresh biomass, dry biomass, total organic carbon and BGC, derived from the product of seagrass areal extent and LAI, all followed a cyclical pattern more consistent with seagrass areal extent than mean LAI (Fig. 6c, d). The variation in total fresh biomass was equally correlated with seagrass areal extent (*r* = 0.63, *n* = 31, *p* = 0.000009) and mean LAI (*r* = 0.61, *n* = 31, *p* = 0.0002). Total fresh biomass ranged from 12 to 27 Gg, with an average of 18 ± 3 Gg, while dry biomass ranged from 2 to 5 Gg, with an average of 4 ± 1 Gg (Fig. 6c). Total carbon content averaged 5 ± 1 Gg over the 30-year period, while BGC averaged 4 ± 1 Gg. Further trend analysis showed only a − 0.7% change in BGC over 30 years, while a *γ* statistic indicated that 291 years of data would be needed for the current Theil-Sen slope to overcome variability in the data if a trend was present (Table 3).

### Effects of Large-Scale Climate Drivers on Seagrasses in St. Joseph Bay

A multiple linear regression analysis did not reveal a relationship between either ENSO or NAO indices and the temporal oscillation in seagrass extent, LAI, or BGC (Table 4). Further, analysis of Landsat’s mean SST against seagrass extent (Fig. S7) revealed no statistically significant relationship (*r*^2^ = 0.0004, *p* = 0.95).

Four tropical storms (Bonnie, Fay, Claudette, Nestor), one hurricane (Hermine), and one major hurricane (Michael) passed over St. Joseph Bay during the 30-year period of this study, all occurring between 2004 and 2019. Tropical Storm Bonnie passed over St. Joseph Bay on 12 August 2004, but our analysis showed that seagrass extent changed by only 1 km^2^ (4%) between October 2003 (27 km^2^) and October 2004 (26 km^2^) which was less than the uncertainty in total cover (~ 7%). However, a 38% decline in seagrass extent was observed between October 2004 (26 km^2^) and March 2005 (16 km^2^). An 18% decline was observed between August 2007 (22 km^2^) and September 2008 (18 km^2^) following the passing of Tropical Storm Fay on 23 August 2008. Seagrass extent recovered to 21 km^2^ on 9 June 2009, but declined again to 19 km^2^ in 2010, after the passage of Tropical Storm Claudette over St. Joseph Bay on 16 August 2009. Hurricane Hermine reached St. Joseph Bay on 1 September 2016, after which seagrass extent decreased from27 km^2^ in October 2015 to 26 km^2^ in October 2016 and further to 24 km^2^ in November 2017. Between March 2018 and November 2019, seagrass extent decreased by 0.3 km^2^. Two storms impacted St. Joseph Bay during this time: Hurricane Michael (10 October 2018) and Tropical Storm Nestor (19 October 2019). Overall, we found six periods of seagrass decline between 2004 and 2019 that followed the four tropical storms and two hurricanes (one major) that passed over St. Joseph Bay.

## Discussion

### Seagrass Classification Using the DCNN

The strong overall agreement (90–96%) between our Landsat classification and both aerial imagery and high spatial resolution satellite imagery demonstrated that our DCNN algorithm reliably identified seagrass pixels across three Landsat missions to produce a 30-year time series analysis of seagrass extent in St. Joseph Bay. Although we observed no significant temporal trend in seagrass extent or LAI over the 30-year period, there were clear oscillating patterns of variability identified in the time series. However, these patterns of variability were unrelated to ENSO or NAO climate indices, SST or tropical cyclones, suggesting that more localized pressures such as grazing, or water quality, may be responsible for the observed seagrass dynamics in St. Joseph Bay.

Several studies have leveraged Landsat’s historical archive to map seagrass habitats using different classification methods, including object-based image analysis and maximum likelihood classifiers (Dekker et al. 2005; Wabnitz et al. 2008; Lyons et al. 2012; Meyer and Pu 2012; Hossain et al. 2015; Misbari and Hashim 2016; Topouzelis et al. 2018; Borfecchia et al. 2019). However, these studies achieved lower accuracies (46–92.5%; mean of 72.6%) than our DCNN algorithm. Our DCNN algorithm performed better at detecting seagrass presence in the 2013 L8 image than in the 2010 L5 image, likely related to the improved signal-to-noise ratio characteristics and 12-bit quantization of the L8 OLI sensor, which permits improved measurement of subtle variability in surface conditions (Irons et al. 2012). Overall, the DCNN algorithm was able to detect seagrass absence between 94 and 97% of the time but these results are likely biased due to imbalanced class sizes resulting from larger areas of non-seagrass compared to seagrass. Furthermore, while the sensitivity analysis indicated that the choice of hyper-parameters had minimal impact on the DCNN output, the stochastic training process led to variations in predicted seagrass area between 0.5 and 1.5 km^2^ across ten scenes, that may contribute to the temporal variation (± 3.0 km^2^) obtained across the time series (Supplemental information text S4, Figs. S8 and S9). Therefore, with the lack of a statistical change and the 3% change in seagrass extent within the 10% instrument classification error, it is possible that the small change observed in seagrass extent may have been caused by sensor or algorithmic noise.

While no aerial assessments were available, Yarbro and Carlson (2016) and Yarbro et al. (2020) provided seagrass extent (km^2^) for the years 1992, 1993, 2003, 2006, 2015, 2017, and 2019, which were between 6 and 38% higher than our semi-automated, pixel-based estimates of seagrass areal extent from the DCNN for those same years. Their estimates, derived from photointerpreters who delineated polygons of dense and patchy seagrass cover from natural color photographs, are likely higher because the patchy seagrass class included unvegetated areas classified as bare sand by our pixel-based approach (Meehan et al. 2005; Coffer et al. 2020). Further, our seagrass classification for the year 2010 was 43% lower than the classification from FL FWC. The distinction between patchy and continuous seagrass classes in the FL FWC seagrass map was based on the perception of texture rather than by quantifying percent cover within the polygons, so we combined patchy and continuous seagrass polygons into a single class representing seagrass presence and the remaining pixels were reclassified as seagrass absence for comparison with our pixel-based classification maps. Consequently, it is likely that the areal extent of the FL FWC polygons overestimated true seagrass cover. Additionally, the 2010 L5 classification showed a stronger agreement with the WV2 classification than the FL FWC seagrass map as demonstrated by the McNemar test, as both the L5 and the WV2 image were classified using the same deep learning algorithm.

Further, it is important to recognize that analyses based on aerial photointerpretation contain inherent unquantified error and do not represent an absolutely true reference for evaluating other approaches (Congalton 1991; Rutchey and Vilchek 1999; Coffer et al. 2020). Manual classification is often assumed to be correct when compared to semi-automated remote sensing classification, but caution should be taken in making such quantitative comparisons since both approaches rely on different mechanisms for classifying ground cover data and quantifying their respective areas (Rutchey and Vilchek 1999). Moreover, interpreter error can substantially affect results when comparing photointerpretation and remote sensing products for temporal trends (Ward et al. 1997). Although image segmentation techniques have a proven success in seagrass classification and mapping (Wicaksono and Lazuardi 2018; Jeon et al. 2021), our pixel-based classification still demonstrated consistency in classifying a time series of images in a semi-automated and efficacious way with high agreement, compared to traditional photointerpretation methods. Some of the difference between our DCNN and the photointerpreter estimates could also have resulted from the large precision values in comparison to the usually lower recall values, which essentially results in an underestimation of seagrass areal cover from the Landsat imagery.

The Landsat imagery provided suitable spatial resolution to map the relatively large, continuous seagrass meadows of St. Joseph Bay with sufficient accuracy to perform time series analyses, but Landsat may not perform well in areas where seagrass shows considerable patchiness at scales smaller than 900 m^2^, such as along the deep edge and across the intertidal zone. Kaufman and Bell (2020) demonstrated the effect of fine-scale versus broad-scale seagrass mapping, suggesting 53% less seagrass mapped using fine-scale approaches, but Hill et al. (2014) found that coarsening the spatial resolution of hyperspectral imagery from1 to 10 m underestimated seagrass area by only 8% and further demonstrated that the effect of scale on seagrass area retrieval depended on the size and number of patches in the scene. Similarly, comparison of the coincident L5 and WV2 classification and L8 and WV2 classification indicated that Landsat’s coarser spatial resolution underestimated seagrass area by 26% and 3% respectively compared to WV2’s 2-m resolution. Compared to L5 and L8, WV2 offers high spatial resolution (1.84 mat nadir, resampled to 2 min level 1B imagery). This difference suggests that the increased spatial resolution in the WV2 imagery may have contributed to the additional detection of small seagrass patches not detected by Landsat (Kovacs et al. 2018). Additionally, our WV2 classification exhibited a higher recall than precision (Table 2), essentially increasing the seagrass extent in comparison to the Landsat-based estimate.

### Temporal Assessment of Seagrass Extent, LAI, and BGC

Our estimated mean seagrass area of 23 ± 3 km^2^ across the time series was consistent with the estimate of 24 km^2^ derived from hyperspectral imagery in 2006 (Hill et al. 2014). Our estimate was also similar to a 1972 estimate of 25.6 km^2^ derived from aerial photography (McNulty et al. 1972) and a 1984 estimate of 23 to 24 km^2^ derived from a multispectral airborne sensor (Savastano et al. 1984). The results in our study show that seagrass meadows have been temporally stable in St. Joseph Bay for the 30-year period, albeit punctuated by high-frequency interannual variability, suggesting rapid recovery following periodic disturbances.

In contrast to the long-term stability of the seagrass populations in St. Joseph Bay, studies of the long-term worldwide temporal dynamics of seagrass have documented declines and losses due to direct anthropogenic disturbances (Tomasko et al. 2005; Thorhaug et al. 2017), hurricane-related impacts (Cho et al. 2017; Challener et al. 2019; León-Pérez et al. 2020), grazing by sea urchins and green turtles (Challener et al. 2019; León-Pérez et al. 2020; Rodriguez and Heck 2020 2021), and climate change (Orth et al. 2006; Lyons et al. 2012; Short et al. 2016) in other locations. While seagrass extent was assessed as stable in St. Joseph Bay, propeller scarring remains a chronic problem, especially in the southern portion of the Bay (Yarbro and Carlson 2016). Such scars, however, are not distinguishable with Landsat’s 30-mresolution but can be identified in higher spatial resolution WV2 imagery using a DCNN approach similar to the one used here for seagrass classification (Hoque et al. 2018).

Hurricanes can have direct, physical impacts on seagrasses, causing sediment deposition, defoliation and/or uprooting (van Tussenbroek et al. 2008), as well as longer term impacts such as light limitation from persistent water column turbidity resulting from heavy rainfall and runoff (Ridler et al. 2006; Steward et al. 2006; Tomasko et al. 2020). Alternating patterns of seagrass decline and growth have also been observed in response to mechanical hurricane damage and recolonization, possibly from storm-induced dispersal seeds and vegetative propagules, as well as reduced herbivore pressure (Creed et al. 2003; León-Pérez et al. 2020). However, even a direct hit by a Category 5 hurricane does not always result in significant damage to healthy seagrass meadows (Dierssen et al. 2003).

We found six seagrass declines between 2004 and 2020 which followed the four tropical storms and two hurricanes (one major) that passed over St. Joseph Bay. Unfortunately, the coarse temporal coverage of images between these storms prevents us from assessing the impacts in greater detail. While most storms produced only a decline in seagrass area within the 2 to 5% uncertainty in our area estimates, the larger decline observed in seagrass area between 2004 and 2005 may have been due to longer-term impacts related to storm run-off from tropical storm Bonnie. The St. Andrew Bay watershed is the only major estuarine drainage basin entirely within the Florida Panhandle, and observations of CDOM collected from the Moderate Resolution Imaging Spectroradiometer (data not shown) indicated that storm runoff from this watershed may have been advected along the coast towards St. Joseph Bay and impacted seagrass meadows there following tropical storms and hurricane events. Long-term runoff-related impacts from hurricane events caused pronounced declines in seagrass abundance in the more eutrophic habitats of Tampa Bay, Charlotte Harbor, and Sarasota Bay in West Florida (Fig. 1; Carlson et al. 2010). Major Hurricane Michael in October 2018 generated waves and currents strong enough to bisect the peninsula adjacent to St. Joseph Park, creating a large passage from St. Joseph Bay into the Gulf of Mexico. Senne (2020) found that seagrass area in the Bay declined between 2017 and 2019 due to a combination of stressors including Hurricane Michael, boat propeller scarring, sea urchin grazing, nutrient runoff, and decreased water quality. In line with Senne (2020), our findings not only confirm the decline in seagrass extent between 2017 and 2019, but also showed a longer decline period starting in 2015. Our findings highlight the use of satellite imagery as a complement to the aerial imagery used in Senne (2020) to better understand drivers of observed changes in seagrass extent. While these stressors may have been responsible for the decline in seagrass area after Hurricane Michael and Tropical Storm Nestor in 2019, our results also showed that seagrass rebounded in March 2020. Such rapid recovery was also observed during the previous years following storm and hurricane impacts. The time series analysis provided by our study can forma fundamental data set for examining future trends in seagrass and carbon dynamics, as related to environmental and climate drivers as well as grazing pressures.

Several studies have reported large-scale declines in seagrass abundance related to changes in the NAO (Murdoch et al. 2007; Pergent et al. 2015) or ENSO indices (Carlson et al. 2018; Lin et al. 2018; Holbrook et al. 2020; Belarmino et al. 2021). Other studies have found that higher seagrass density and biomass were related to increased flushing of nutrients into a temperate Pacific estuary from the rainfall associated with a La Niña event in Washington, USA (Thom et al. 2003). Two major ENSO events during our time series, 1997–1998 and 2004–2005, corresponded to the two lowest seagrass areas as well as low fresh biomass and BGC values. The highest LAI was recorded for 1998, while an average LAI was recorded for 2005. Nevertheless, we observed no correlation between the interannual variability in seagrass extent, LAI and BGC in St. Joseph Bay and ENSO, NAO, and surface water temperature. Thus, it appears that seagrass communities in St. Joseph Bay may be buffered from disruptions in atmospheric and oceanic circulation and weather patterns associated with such events, perhaps because there is no direct watershed drainage into the system, a unique situation relative to most estuaries and coastal lagoons on the Gulf and Atlantic coasts of the USA. St. Joseph Bay is the only body of water in the eastern Gulf of Mexico not influenced by the inflow of freshwater (DEP 2008), with all the freshwater going into Apalachicola Bay, which is directly adjacent to St. Joseph Bay.

The Gulf Coast falls within a moderate energy coastal area (Tanner 1959), with average breaker heights of 0.1 to 0.5 m. Waves traveling northward through the Gulf of Mexico are refracted clockwise around the Cape San Blas shoals in such a manner as to arrive nearly parallel to the beach. In general, the currents in St. Joseph Bay sweep around the St. Joseph Peninsula and a cyclonic circulation pattern occurs in the central portion of the bay (DEP 2008). Current movement occurs on the surface throughout a major portion of the bay, diminishing rapidly below the 2-mdepth contour. In most of the extensive shallow reaches of the southern end of the bay, there is no appreciable current except for the daily tide. Therefore, this most productive area of the bay functions largely as a closed system(Stewart 1962), which could explain why the seagrass communities in St. Joseph Bay may be protected from disruptions in atmospheric and oceanic circulation and weather patterns associated with such events.

The seagrass declines reported here were also followed by rapid recovery producing a stable mean value with no temporal trend, suggesting that rather than large-scale climate patterns, more localized drivers such as meadow structure, hydrodynamic and physical setting, or grazing pressure could control the interannual growth/decline and recolonization potential after disturbance or loss (Lyons et al. 2013). As a matter of fact, Rodriguez and Heck (2021) reported that grazing pressure from green turtles has been rising in St. Joseph Bay while Challener et al. (2019) reported an increased abundance of sea urchins in St. Joseph Bay and their remarkable resilience to the impact of Hurricane Michael. Therefore, it is possible that herbivory from green turtles and sea urchins may be contributing to the patterns observed in seagrass dynamics of St. Joseph Bay. Additionally, our seagrass frequency maps have shown that variability in seagrass extent occurred mostly in the intertidal region and along the deep edges of seagrass distribution. In the intertidal region, this is possibly due to desiccation where the seagrasses are exposed at low tide. Desiccation is usually the limiting factor controlling the upper limit of seagrass growth on the intertidal flat (Van Katwijk and Hermus 2000; Van der Heide et al. 2010), although this may change with rising sea levels. Seagrass variability along the deep edges may be a result of hydrodynamic forces (Uhrin and Turner 2018) or deepening of the water column with not enough light getting to seagrasses (Pattarach et al. 2018).

Our calculations of LAI, biomass, and BGC employed a previously validated satellite-based technique developed specifically for St. Joseph Bay, and a series of transfer coefficients to retrieve bottom reflectance in the green band and derive LAI, biomass, and BGC (Hill et al. 2014). Our LAI estimates, based on the 30-mspatial resolution imagery of Landsat 5–8, were consistent with the value reported by Hill et al. (2014) from hyperspectral, 1-m spatial resolution imagery. Additionally, the lack of a statistical difference between in situ LAI and L5-retrieved LAI for St. Joseph Bay indicated that optical LAI retrieval from Hill et al. (2014) provides reliable estimates of LAI from multispectral remote sensing imagery. The higher LAI values reported in June 1998, despite a low seagrass area, are consistent with reports of increased leaf production rates during the summer (Kaldy and Dunton 2000). The time series analysis showed that changes in LAI were not driven by changes in seagrass area. In fact, seagrass area declined in 1998, but LAI (and BGC) increased. This implies that factors controlling seagrass LAI may be somewhat independent of those controlling seagrass extent in this system. System wide reductions in seagrass extent seem to be the result of loss along deep edges and in the intertidal areas with low LAI while the well-established, dense seagrass meadows with higher LAI and BGC remained stable.

Although seagrass communities in St. Joseph Bay and their blue carbon appeared stable over the 30-year period and relatively unaffected by climate disruptions and tropical cyclones, forecasts suggest oceans will continue to warm and tropical cyclones will continue to intensify (Pachauri et al. 2014; Babcock et al. 2019; Ainsworth et al. 2020). Thus, our study demonstrates how a consistent, long-term time series of Bay-wide seagrass distribution derived from satellite imagery can provide a basis against which future change in the system can be quantified in terms of water quality or climate threats (e.g., carbon emissions from seagrass degradation and loss, nutrient pollution, surface water warming, acidification, sea-level rise, extreme events, etc.). Further, our analysis provides a proof of concept for using time series analysis of remote sensing observations in seagrass ecology. The semi-automated approach presented here allowed for reproducible and expeditious seagrass mapping with better spatial and temporal resolution compared to traditional mapping techniques, which is critical for understanding seagrass dynamics. Thus, our study importantly demonstrates that remotely sensed time series analyses provide an excellent complement to ground monitoring efforts, enabling a holistic approach to seagrass ecology research.

Although the present study provided insights into the long-term dynamics of seagrass in St. Joseph Bay, future research should examine seagrass beds at a higher temporal frequency to identify how both natural and anthropogenic stressors (e.g., wave energy exposure and frequency, nutrient inputs, herbivores abundance) relate to intra-annual changes in seagrass extent and their carbon storage. While we have achieved consistency in training samples by using the same ROI locations across all images in the time series and excluding the optically-deep water class across all Landsat images, research efforts are currently underway to achieve consistency in processing of tides and bathymetry across multiple locations for seagrass ecosystems across the USA, which would provide a powerful tool for examining synoptic seagrass dynamics. Once optimized, the methods and trends investigated in this study would be applicable and relevant to other locations that may require broad scale, retrospective mapping of seagrass, and help address the uncertainties regarding regional estimates of seagrass areal extent and carbon storage.

## Supplementary Material

Supplement1

## Figures and Tables

**Table 1:** Number of polygons and range of pixel counts for regions of interest defined for each class

Class	Number of polygons	Pixel count per polygon (range)

Optically-deep water	3	2394–2640
Sand	5	60–256
Seagrass	6	77–726
Land	3	440–4176
Intertidal	3	77–126

**Table 2:** Agreement metrics for seagrass absence and presence, derived from a deep convolutional neural network (DCNN) classification of Landsat 5 (L5) and Landsat 8 (L8) imagery. The L5 classification from 2010 was compared to the 2010 Florida Fish and Wild-life Conservation Commission (FL FWC) survey as well as a DCNN classification of a coincident WorldView-2 (WV2) image. The L8 classification from 2013 was compared to the DCNN classification of a coincident WV2 image

		Precision	Recall	*F*-measure	Overall accuracy	*K*

L5-FL FWC	Absence	0.89	0.99	0.94	0.90	0.62
	Presence	0.93	0.52	0.67		
L5-WV2	Absence	0.91	0.97	0.94	0.90	0.64
	Presence	0.77	0.54	0.63		
L8-WV2	Absence	0.97	0.98	0.97	0.96	0.95
	Presence	0.88	0.85	0.87		

**Table 3:** Summary of the seasonal Mann–Kendall and Theil-Sen slope statistic results for the time series of change in seagrass area, leaf area index (LAI) and belowground organic carbon (BGC) between 1990 and 2020. Statistics include the number of scenes (*n*), Theil-Sen slope, percent change per year, Kendall’s *τ*, *p* value, and the *γ* statistic. The Theil-Sen slope is used as the denominator in computing *γ*, thus, a slope value of 0 for LAI prevents the computation of *γ*

	*n*	Slope	Percent change	*τ*	*p*	*γ*

Seagrass area (km^2^)	31	0.03	1.7%	0.09	0.59	76
LAI	31	0	0%	− 0.13	0.42	Undefined
BGC (kg/Bay)	31	− 2305	− 0.7%	− 0.01	1	291

**Table 4:** Multiple linear regression model summary for the time series of change in seagrass area, leaf area index (LAI), and belowground organic carbon (BGC) between 1990 and 2020, as related to the climate indices El Niño Southern Oscillation (ENSO) and North Atlantic Oscillation (NAO). Statistics include the number of scenes (*n*), the *β* coefficient, which measures the association between the predictor variable (ENSO and NAO) and the outcome (seagrass area, LAI, BGC), the correlation coefficient (*r*^2^), the t-statistic (*t*), which evaluates whether the *β* coefficient is significantly different from zero and the *p* value, which indicates whether the predictor variables are significantly correlated to the outcome variables

	*n*	Variable	*β* coefficient	*r* ^2^	*t*	*p*

Seagrass area (km^2^)	31	(Intercept)	23.3	0.10	50.8	
		ENSO	0.92		1.93	0.06
		NAO	0.39		0.83	0.41
LAI	31	(Intercept)	1.59	− 0.02	43.7	
		ENSO	0.03		1.05	0.30
		NAO	− 0.02		− 0.57	0.57
BGC (kg/Bay)	31	(Intercept)	3,825,141	0.13	33.3	
		ENSO	300,476		2.51	0.02
		NAO	− 13,488		− 0.11	0.91
