# Supplementary material for: Temporal Stability of Seagrass Extent, Leaf Area, and Carbon Storage in St. Joseph Bay, Florida: a Semi-automated Remote Sensing Analysis
Source: Estuaries Coast. Author manuscript; Available in PMC 2023 Nov 1. (PMC10054859; doi:10.1007/s12237-022-01050-4)
Supplement: Supplement1 [file NIHMS1841016-supplement-Supplement1.pdf]

Electronic Supplemental Material

Temporal Stability of Seagrass Extent, Leaf Area and Carbon Storage in St. Joseph Bay, Florida: A Semi-Automated Remote Sensing Analysis

Marie Cindy Lebrasse[1,2*], Blake A. Schaeffer[3], Megan M. Coffer[1], Peter J. Whitman[1], Richard C. Zimmerman[4], Victoria J. Hill[4], Kazi A. Islam[5], Jiang Li[5] and Christopher L. Osburn[2]

Submitted to *Estuaries and Coasts: 6 January 2021*

[1]ORISE Fellow, U.S. Environmental Protection Agency, Office of Research and Development, Durham, North Carolina, USA

[2]Department of Marine, Earth and Atmospheric Sciences, North Carolina State University, Raleigh, North Carolina, USA

[3]U.S. Environmental Protection Agency, Office of Research and Development, Durham, North Carolina, USA

[4]Department of Ocean and Earth Sciences, Old Dominion University, Norfolk, Virginia, USA

[5]Department of Electrical and Computer Engineering, Old Dominion University, Norfolk, Virginia, USA

*Corresponding Author: lebrasse.marie@epa.gov Tel: 919-541-5612

Keywords:

seagrass; Submerged Aquatic Vegetation; leaf area index; carbon; Landsat; trend analysis

Table S1. Definition of acronyms used throughout the manuscript

Acronym	Definition
**BGC**	Belowground organic carbon
**CDOM**	Chromophoric dissolved organic matter
**Chl *a***	Chlorophyll *a*
**DCNN**	Deep convolutional neural network
**DEM**	Digital elevation model
**DOS**	Dark object subtraction
**ENSO**	El Niño-Southern Oscillation
**FL FWC**	Florida Fish and Wildlife Conservation Commission
K_d	Spectral diffuse attenuation coefficient
K_{Lu}	Upwelling diffuse attenuation coefficient
**L1TP**	Landsat collection-1 Level-1 Terrain and Precision data
**L5**	Landsat 5 Thematic Mapper (TM)
**L7**	Landsat 7 Enhanced Thematic Mapper (ETM+)
**L8**	Landsat 8 Operational Land Imager (OLI)
**LAI**	Leaf area index
**NAO**	North Atlantic Oscillation
R_b	Bottom reflectance in the green band
R_{rs}	Remote sensing reflectance
**ROI**	Region of interest
**VBS**	University of South Florida Virtual Buoy System
**WV2**	WorldView-2

Table S2. Image Information for Landsat 5 (L5) thematic mapper (TM), Landsat 7 (L7) enhanced thematic mapper (ETM+) and Landsat 8 (L8) operational land imager (OLI). St. Joseph Bay, Florida is located in Landsat path 19 and row 39. All images were obtained from USGS Earth Explorer.

Sensor	Filename	Acquisition Date	Overpass time (UTC)	Cloud Cover (%)	Sun Elevation (º)	Sun Azimuth (º)
**L5 TM**	LT05_L1TP_019039_19901027_20160930_01_T1	27 Oct 1990	15:33	0.00	39.0	143.5
	LT05_L1TP_019039_19910912_20160929_01_T1	12 Sep 1991	15:37	1.00	51.3	126.4
	LT05_L1TP_019039_19920914_20160928_01_T1	14 Sep 1992	15:35	6.00	50.4	127.4
	LT05_L1TP_019039_19931003_20160927_01_T1	3 Oct 1993	15:35	0.00	45.9	136.3
	LT05_L1TP_019039_19941123_20160926_01_T1	23 Nov 1994	15:27	0.00	31.6	146.4
	LT05_L1TP_019039_19951110_20160926_01_T1	10 Nov 1995	15:13	6.00	32.8	141.5
	LT05_L1TP_019039_19961027_20160924_01_T1	27 Oct 1996	15:33	2.00	39.0	143.9
	LT05_L1TP_019039_19971115_20160923_01_T1	15 Nov 1997	15:47	8.00	35.6	150.8
	LT05_L1TP_019039_19980627_20160922_01_T1	27 Jun 1998	15:51	0.00	63.8	98.6
	LT05_L1TP_019039_19991105_20160919_01_T1	5 Nov 1999	15:49	4.00	38.5	149.9
	LT05_L1TP_019039_20001022_20160918_01_T1	22 Oct 2000	15:52	2.00	42.6	147.8
	LT05_L1TP_019039_20011110_20160917_01_T1	10 Nov 2001	15:53	0.00	37.4	151.7
	LT05_L1TP_019039_20021129_20160916_01_T1	29 Nov 2002	15:45	0.00	32.3	150.7
	LT05_L1TP_019039_20031015_20160914_01_T1	15 Oct 2003	15:51	0.00	44.8	145.3
	LT05_L1TP_019039_20041001_20160913_01_T1	1 Oct 2004	15:57	5.00	49.5	142.4
	LT05_L1TP_019039_20050902_20160912_01_T1	2 Sep 2005	16:01	2.00	57.4	128.8

	LT05_L1TP_019039_20061124_20160908_01_T1	24 Nov 2006	16:08	0.00	35.5	156.6
	LT05_L1TP_019039_20070807_20160907_01_T1	7 Aug 2007	16:07	6.00	63.1	115.4
	LT05_L1TP_019039_20080926_20160905_01_T1	26 Sep 2008	15:58	0.00	50.9	140.3
	LT05_L1TP_019039_20090609_20160905_01_T1	9 Jun 2009	16:01	2.00	66.7	102.3
	LT05_L1TP_019039_20101119_20160831_01_T1	19 Nov 2010	16:03	8.00	36.2	155.2
	LT05_L1TP_019039_20111005_20160830_01_T1	5 Oct 2011	16:01	0.00	49.0	145.0
**L7 ETM+**	LE07_LITP_019039_20121031_20160910_01_T1	31 Oct 2012	16:09	1.00	41.7	155.0
**L8 OLI**	LC08_L1TP_019039_20131026_20170308_01_T1	26 Oct 2013	16:15	0.01	43.8	155.8
	LC08_L1TP_019039_20141114_20170303_01_T1	14 Nov 2014	16:13	0.01	38.3	157.8
	LC08_L1TP_019039_20151016_20170225_01_T1	16 Oct 2015	16:13	0.77	46.9	152.7
	LC08_L1TP_019039_20161002_20170220_01_T1	2 Oct 2016	16:13	8.07	51.1	148.3
	LC08_L1TP_019039_20171106_20171121_01_T1	6 Nov 2017	16:13	4.97	40.4	157.1
	LC08_L1TP_019039_20180314_20170320_01_T1	14 Mar 2018	16:13	0.01	49.8	140.1
	LC08_L1TP_019039_20191128_20191128_01_RT	28 Nov 2019	16:13	0.7	35.2	158.2
	LC08_L1TP_019039_20200319_20170326_01_T1	19 Mar 2020	16:13	3.12	51.9	138.7

Table S3. A contingency table of the McNemar test results to determine the statistical difference between agreement assessments of the 2010 L5 classification and the 2010 Florida Fish and Wildlife Conservation Commission (FL FWC) survey (L5-FWC) as well as the 2010 L5 classification and a DCNN classification of a coincident WorldView-2 (WV2) image (L5-WV2).

		**L5-WV2**	
		Agree	Disagree
**L5-FWC**	Agree	158892	7209
	Disagree	8512	10851

S1. Detailed description of satellite image processing

Following Coffer et al. (2020), the median of the lowest 5% of the NIR distribution was used to characterize atmospheric contamination while preserving true water-leaving reflectances. Iterations of the Rayleigh exponent (4.0, 4.5 and 4.75) were tested to achieve sufficient spectral separation within the image while maintaining expected spectral responses and avoiding overcorrecting the image to the point where pixel values become negative. A Rayleigh exponent of 4.75 for both L5 and L7 and a Rayleigh exponent of 4.0 for L8 imagery created enough spectral separation while maintaining the expected R_{rs} amplitudes and spectral shapes necessary to distinguish between the five classes in St. Joseph Bay (Fig. S1).

S2. Evaluation of the efficacy of the DOS method on Landsat imagery

Implementation of the DOS approach through our standardized process required optimization of the Rayleigh exponent. A Rayleigh exponent of 4.75 provided the necessary atmospheric correction in shorter wavelengths for both L5 and L7 while a Rayleigh exponent value of 4.0 was found to optimize the DOS approach for the atmospheric correction of L8 imagery. These Rayleigh exponents created enough spectral separation while maintaining the expected R_{rs} amplitudes and spectral shapes necessary to distinguish between the five classes in St. Joseph Bay (Fig. S1). In comparing the spectral and spatial ability of L8 and WV2 to distinguish seagrass, their remote sensing reflectance values were within 13% ± 27% across all bands (Fig. S2). Despite an offset in the wavelength values for each band used from L8 and WV2, all classes displayed similar spectral shapes for both sensors, increasing confidence in similar performance of the DOS correction method for Landsat and WorldView.

Previous studies have shown that relatively high Rayleigh exponents were more appropriate to correct for the selective scattering that occurs in shorter wavelengths during clear conditions and low Rayleigh exponents were more appropriate in hazy conditions where scattering is more uniform across all wavelengths (Curcio, 1961; Chavez, 1988). Despite similarly clear conditions in most of our L5, L7 and L8 scenes, a higher Rayleigh exponent was used for L5 and L7 compared to L8. This suggests that the optimal Rayleigh exponent for the images used in this study is more dependent on variability between sensors than the variability in atmospheric scattering between scenes. Such variability may be attributable to L8’s narrower spectral bands compared to L5 and L7, which reduces the atmospheric absorption and also the sensitivity to atmospheric changes in terms of water vapor content, providing more equal scattering across wavelengths (Irons et al. 2012).

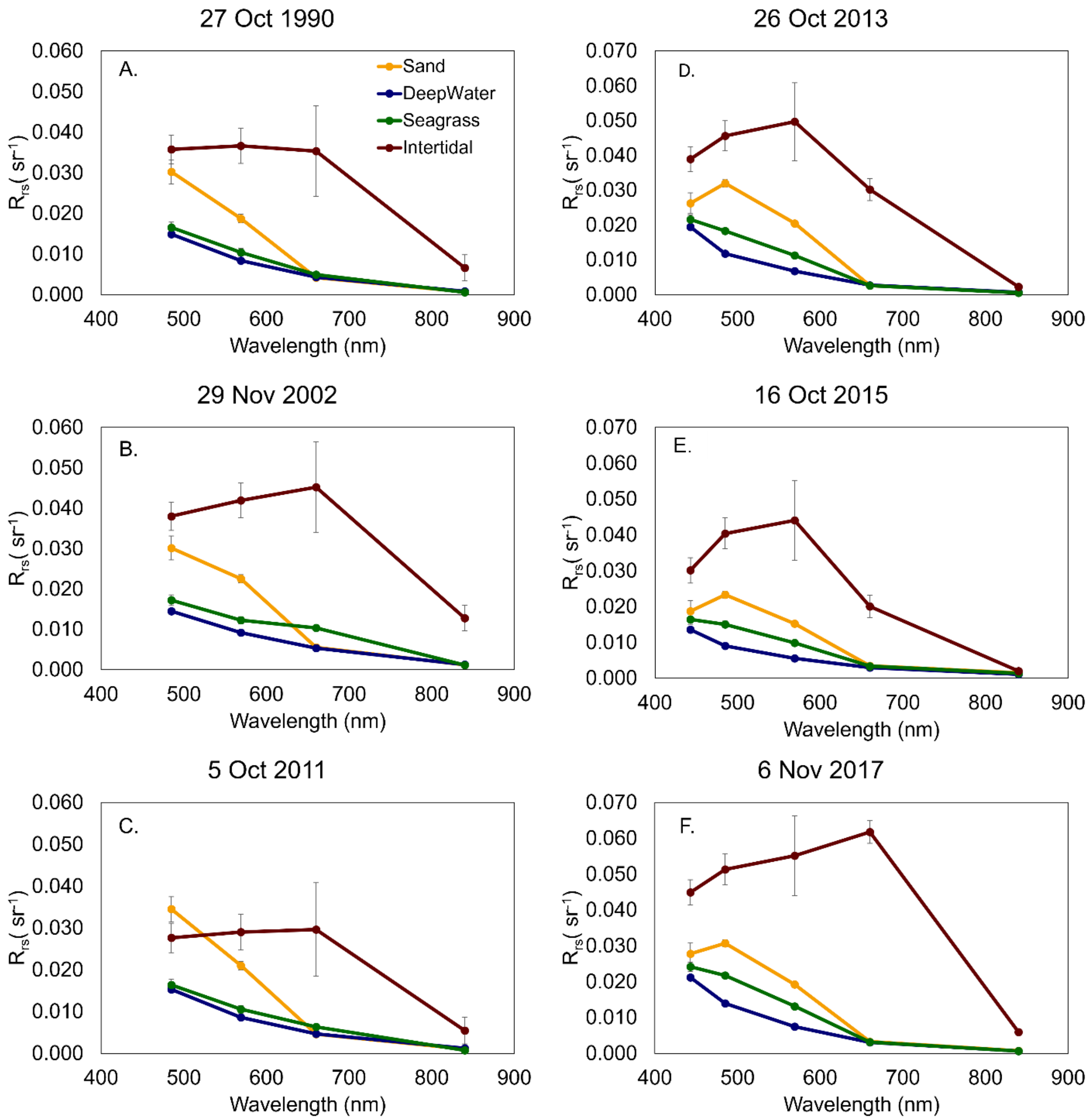

Figure S1. Comparison of the spectral signatures of each class for a subset of images for L5 (A-C) and L8 (D-F) using the DOS approach. For each band, the average and standard deviation of remote sensing reflectance were derived from a ROI drawn for each class. ROIs preserved the same location across all images in both sensors. ROIs were also generated for land but are not shown here.

Further inspection of the reflectance spectra for the different classes between the coincident L8-WV2 scene revealed similar spectra between both sensors and comparison of their classification featured only a 3% difference in seagrass area mapped in each image. While other studies have shown good performance of the DOS method for inland and coastal water applications using one sensor, including for seagrass mapping (Wicaksono and Hafizt, 2017; Wicaksono and Lazuardi, 2018; Traganos et al. 2018; Thalib et al. 2019; Kohlus et al. 2020), results from our study indicated that the classification algorithm performed similarly across different sensors given their similar spectral shapes (Fig. S2), reinforcing the suitability of the DOS method to correct both medium-(L8) and high-(WV2) resolution satellite imagery to spectrally distinguish seagrass in the Bay.

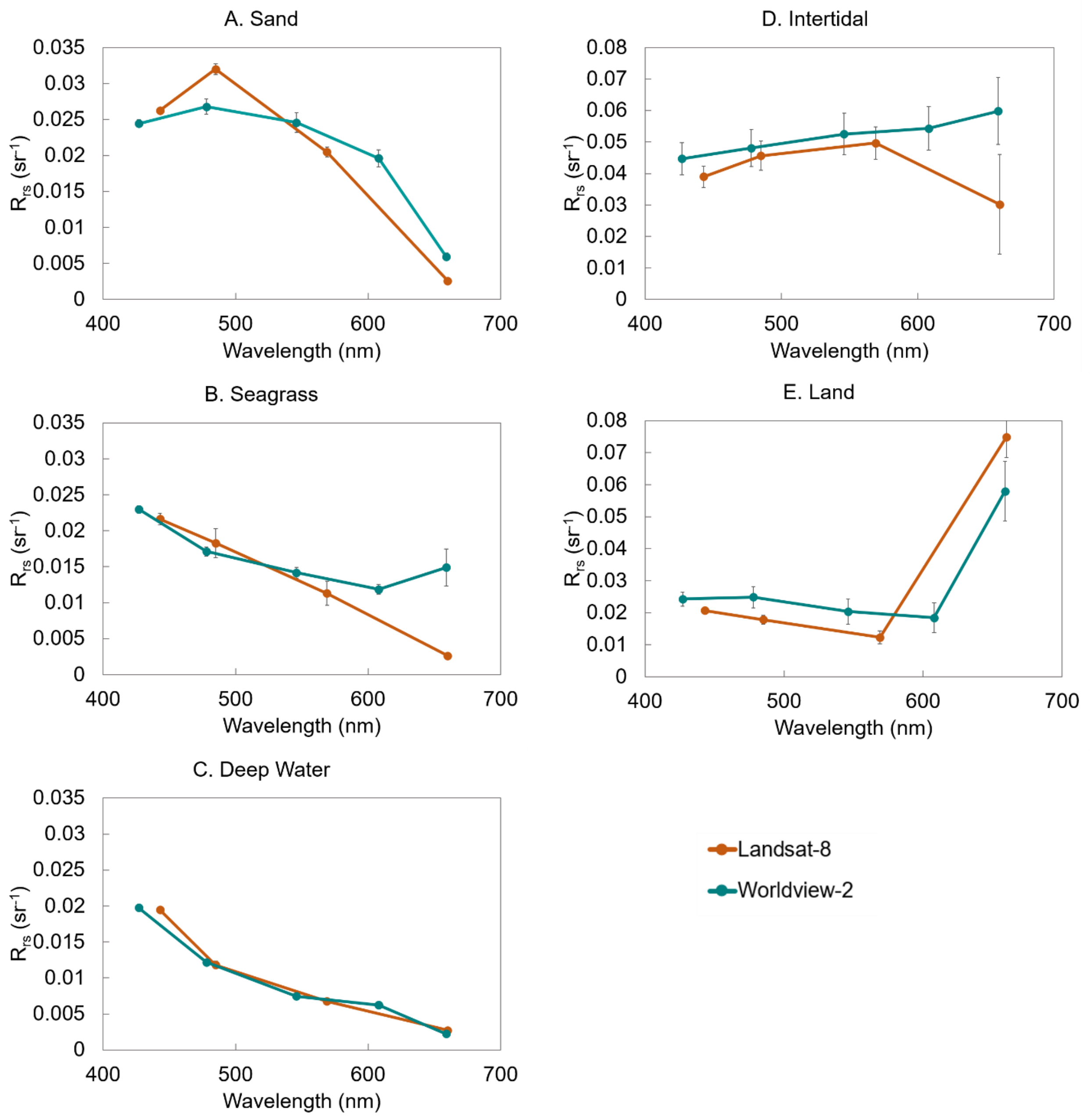

Figure S2. Comparison of the spectral signatures of A. sand, B. seagrass, C. deep water, D. intertidal and E. land for a L8 image (26 October 2013) and a WV2 image (24 October 2013), corrected using the DOS approach. Each plot represents the mean remote sensing reflectance for the class, with the error bars representing 1 standard deviation (SD) for each band. The mean

reflectance for each class was derived from a ROI drawn for that class at the same location in both images.

S3. Detailed description of the deep convolutional neural network

A DCNN with six sequential layers was trained with the ROI training sets using the Keras package in Python 3.5 (Python Core Team, 2015). The final layer of the DCNN leveraged a SoftMax activation function to produce a pixel-wise conditional probability estimate for each class. Each pixel was then assigned to the class with the highest probability estimate. The model was trained for 500 epochs with cross-entropy loss and adaptive moment optimization and applied to the imagery to produce pixel-wise classification maps, based on our sensitivity analysis using filter dimensions of 1x1 and 3x3.

The DCNN model used in this study consists of two convolutional layers, two dropout layers, one flatten layer and one SoftMax layer. The first convolutional layer uses 32 filters with 1*1*7 (number of bands of Landsat) kernel size followed by a dropout layer with 0.01 probability. The next convolutional layer has 16 filters with 3*3*32 kernel size. Then, we use a dropout layer with 0.01 probability and a flatten layer to make it a vector of size 16. Finally, we applied a SoftMax layer to perform classification in the last layer.

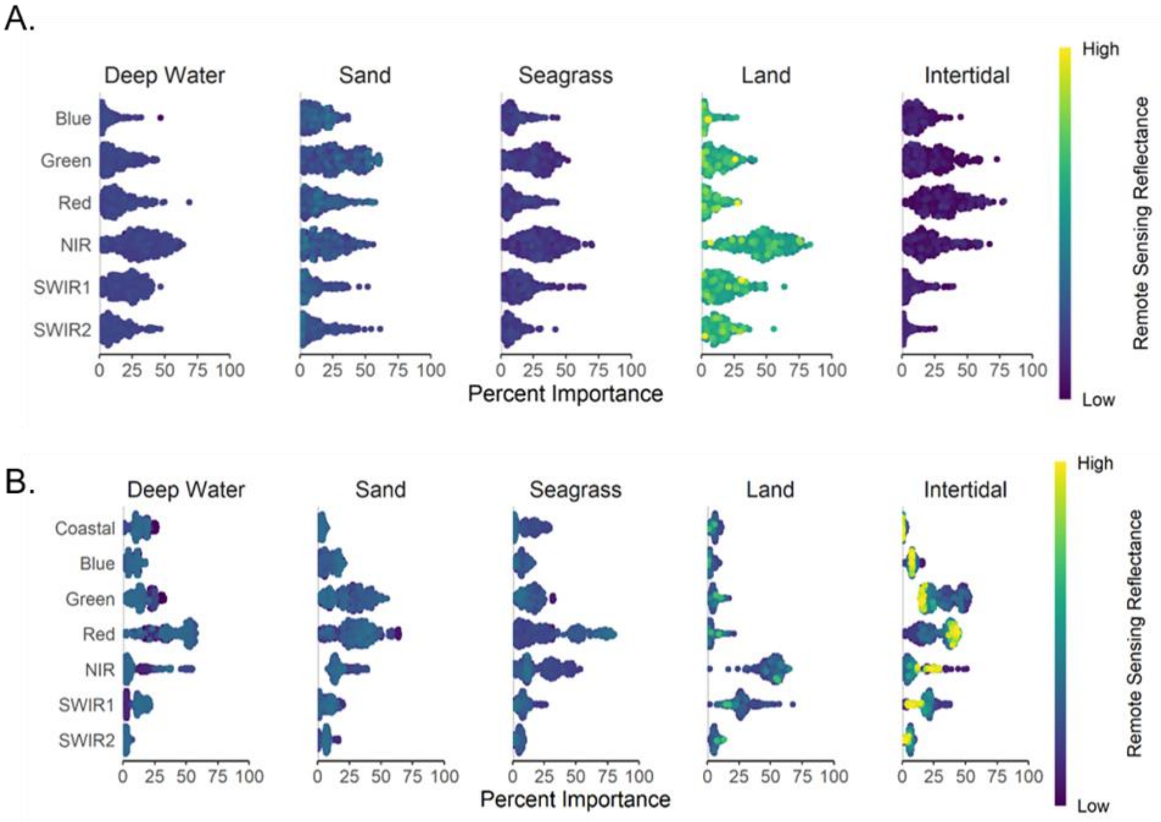

Figure S3. SHAP values for each of (a) Landsat 5 band's contribution and (b) Landsat 8 band's contribution to the DCNN classification of optically-deep water, sand, seagrass, land and intertidal class. The green, the red and the NIR band appear to have a greater impact on each of the five classes classification.

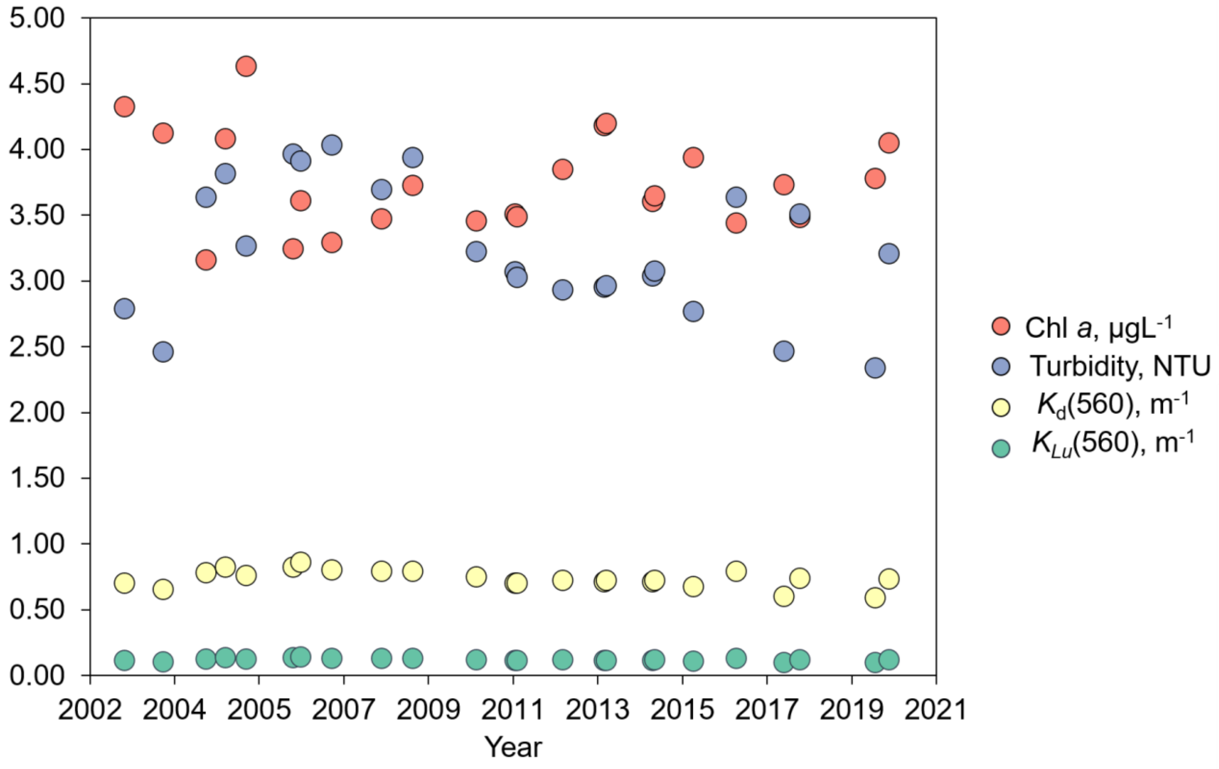

Figure S4. Time series of chlorophyll *a* (Chl *a*) and turbidity retrieved from the Virtual Buoy System (VBS) and $K_d(560)$ and K_{Lu} calculated from the GrassLight model (Zimmerman et al. 2015) based on these optical conditions between 2002 and 2020. Chl *a* and turbidity oscillate out of phase to stabilize $K_d(560)$ throughout the 18-year period. This demonstrated stability in $K_d(560)$ and K_{Lu} over time allowed us to apply a mean $K_d(560)$ and K_{Lu} in leaf area index (LAI) retrieval using the method outlined in Hill et al. (2014).

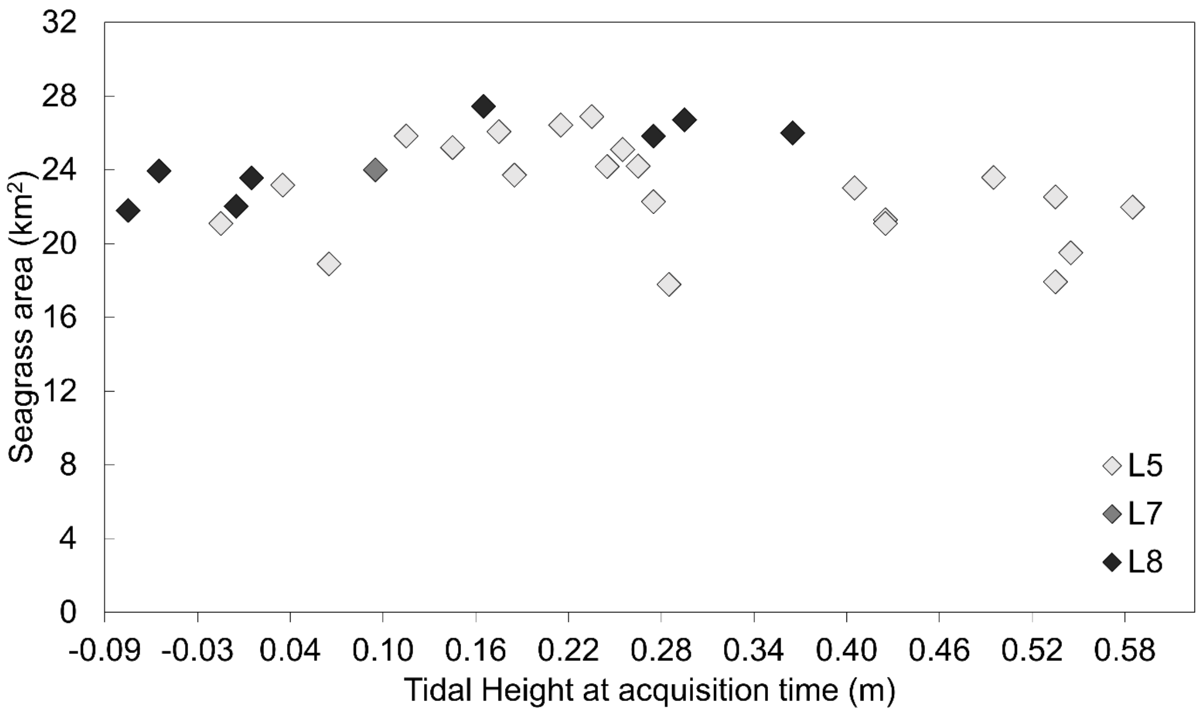

Figure S5. Seagrass surface area as a function of tidal height during image acquisition time for the Landsat 5 (L5), Landsat 7 (L7) and Landsat 8 (L8) scenes.

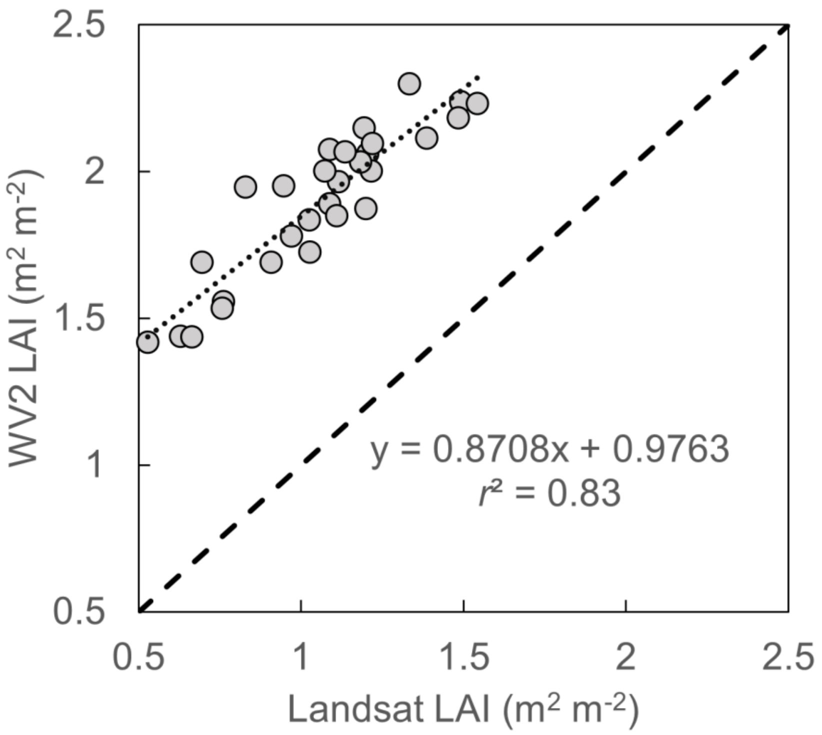

Figure S6. Relationship between Landsat-derived leaf area index (LAI) from a satellite image acquired on 19 Nov 2010 and WorldView-2 (WV2)-derived LAI from a satellite image acquired on (14 Nov 2010). Dotted line represents the 1:1 line.

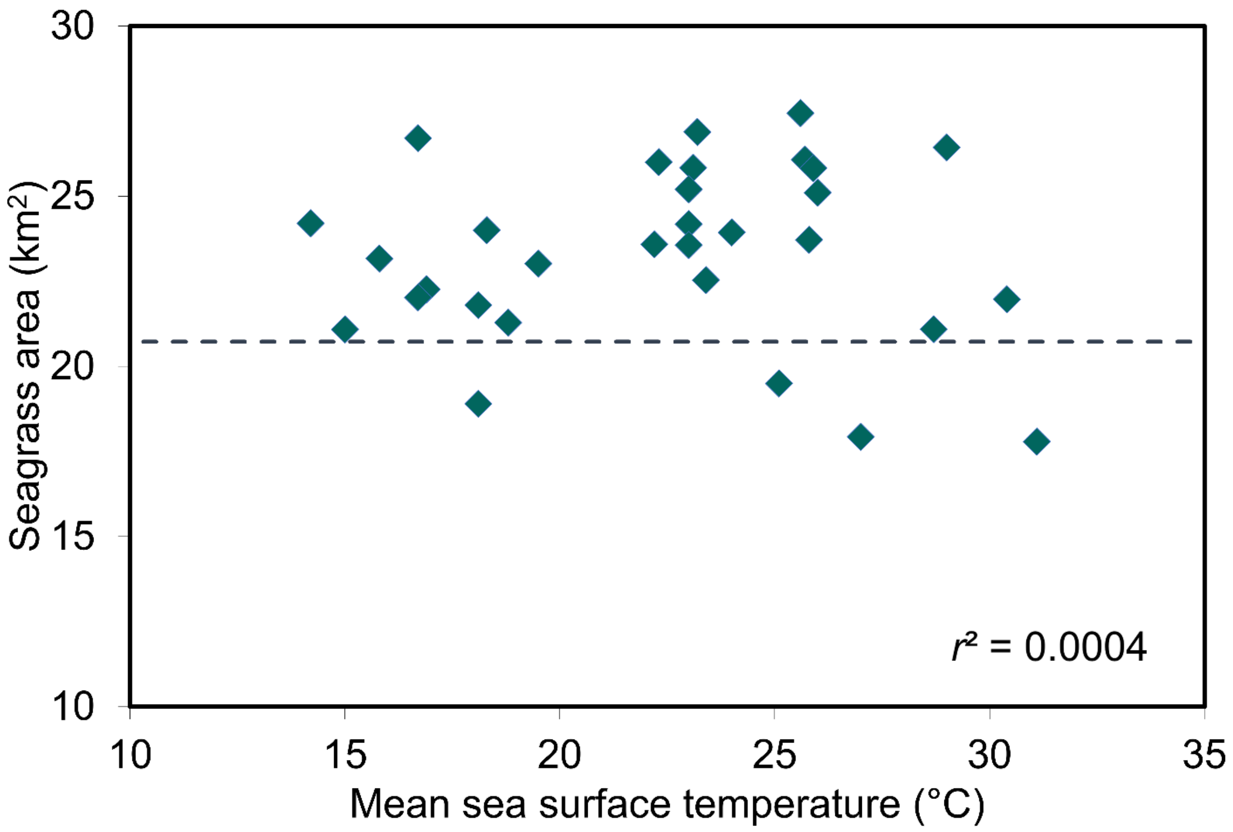

Figure S7. Seagrass surface area as a function of mean sea surface temperature for L5, L7, and L8. The dotted line represents the intercept and the slope was not significantly different from 0.

S4. Sensitivity of seagrass detection to epochs and filter dimensions

The stochastic learning process required to train a DCNN is controlled by hyperparameters. The values of these hyperparameters can impact the accuracy, parsimony and generality of a model, but their optimization is specific to each modeling task. As such, a sensitivity analysis was performed to determine the effects of two hyperparameters—number of epochs and filter dimensions—on predictions of seagrass area within ten Landsat 8 scenes in St. Joseph Bay. The number of epochs defines the number of times the training data (i.e., ROIs) are exposed to the DCNN and the filter dimensions dictate the size of the receptive field where information from the training data are extracted through convolution. In addition to their impact on the aforementioned aspects of model performance, these hyperparameters were chosen for further analysis because they have a large effect on the time required to train a DCNN.

We tested ten combinations of epochs and filter dimensions: epochs of 100, 200, 300, 400 and 500 with filter dimensions of 1x1 and 3x3. The level of uncertainty resulting from stochastic training was also quantified by training ten separate models for each combination of epochs and filter dimensions. The same ROIs were used as training data for each model permutation to ensure that the results highlight variation due to hyperparameters and stochastic training rather than the selection of ROIs. Once the models were trained, they were applied to each of the ten Landsat 8 scenes to produce pixel-wise classifications. In total, there were ten classifications of each scene for each unique combination of epochs and filter dimensions (i.e., 10 combinations of epochs and filter dimensions x 10 permutations x 10 Landsat 8 scenes=1000 classifications) and the pixel-based area of the seagrass pixels was converted to square kilometers.

Fig. S8 shows three classifications of the same scene that were produced by three separate models trained with the same ROIs, filter dimensions and number of epochs. This Figure highlights that when all else is the same, stochastic training can produce variability in results. Variation in predicted seagrass area due to hyperparameters and stochastic training has the potential to obscure a meaningful analysis of how seagrass area varies over time. Fig. S9 provides a time series of predicted seagrass area, averaged across ten permutations with 95% confidence intervals, for each scene and each combination of epochs and filter dimensions. The confidence intervals are small enough, that they are part of the markers on Figure S9. Aside from two scenes, 25 September 2019 and 14 March 2018, the standard deviation of predicted seagrass area for each epoch and filter dimension ranged between 0.5-1.5 km^2. While the predicted seagrass area does vary between combinations of epochs and filter dimensions, this variation is minimal enough that the trends remain similar.

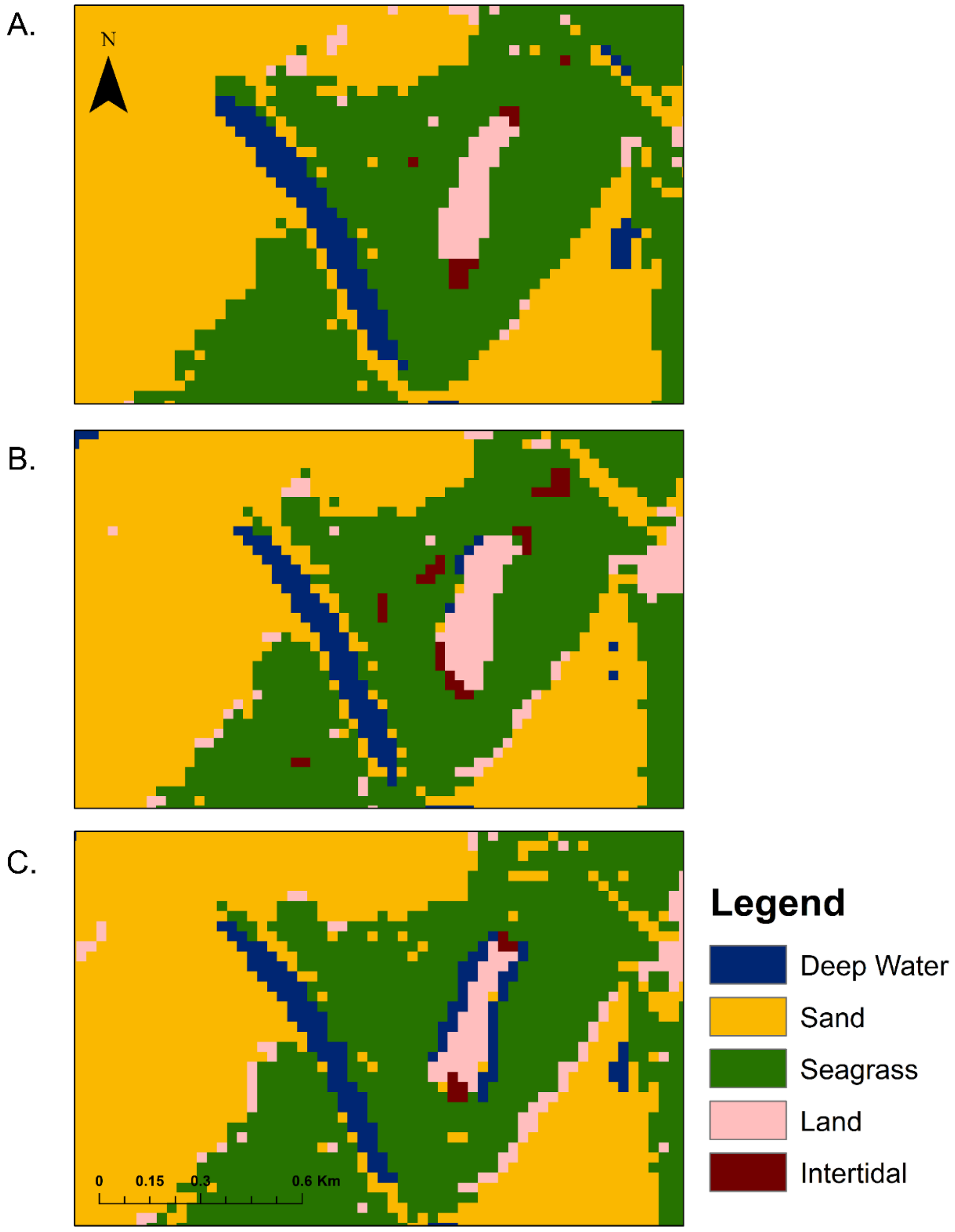

Figure S8. A., B., and C. represent three classifications of a Landsat 8 scene from 10 October 2013. Each classification was produced by a separate model trained with the same training data, epochs, and filter dimensions.

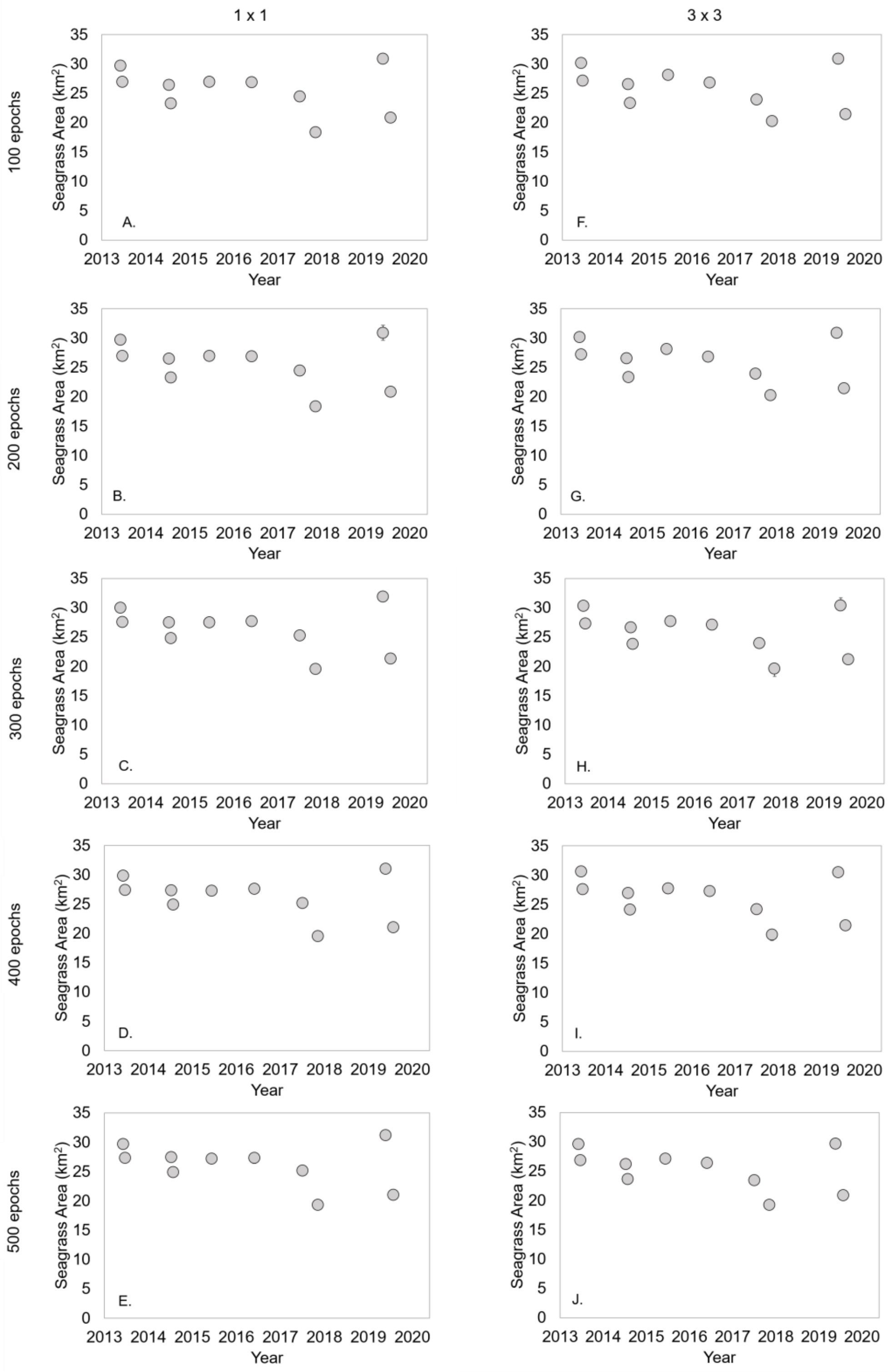

Figure S9. Time series of predicted seagrass area averaged across ten permutations with 95% confidence interval for each scene using a 1×1 filter dimension (A-E) and 3×3 filter dimension (F-J) across five different epochs (100-500).

References

Chavez Jr, P. S. (1988). An improved dark-object subtraction technique for atmospheric scattering correction of multispectral data. *Remote Sensing of Environment*, *24*(3), 459-479.

Coffer, M. M., Schaeffer, B. A., Zimmerman, R. C., Hill, V., Li, J., Islam, K. A. and Whitman, P. J. (2020). Performance across WorldView-2 and RapidEye for reproducible seagrass mapping. *Remote Sensing of Environment*, *250*, 112036.

Curcio, J. A. (1961). Evaluation of atmospheric aerosol particle size distribution from scattering measurements in the visible and infrared. *JOSA*, *51*(5), 548-551.

Kohlus, J., Stelzer, K., Müller, G. and Smollich, S. (2020). Mapping seagrass (Zostera) by remote sensing in the Schleswig-Holstein Wadden Sea. *Estuarine, Coastal and Shelf Science*, 106699.

Irons, J. R., Dwyer, J. L., and Barsi, J. A. (2012). The next landsat satellite: The landsat data continuity mission. *Remote Sensing of Environment*, *122*, 11-21.

Python Core Team, 2015. Python: A Dynamic, Open Source Programming Language. https://www.python.org/.

Thalib, M. S., Faizal, A. and La Nafie, Y. A. (2019). Remote Sensing Analysis of Seagrass Beds in Bontosua Island, Spermonde Archipelago. In *IOP Conference Series: Earth and Environmental Science* (Vol. 253, No. 1, p. 012047). IOP Publishing.

Traganos, D., Aggarwal, B., Poursanidis, D., Topouzelis, K., Chrysoulakis, N. and Reinartz, P. (2018). Towards global-scale seagrass mapping and monitoring using Sentinel-2 on Google Earth Engine: The case study of the aegean and ionian seas. *Remote Sensing*, *10*(8), 1227.

Wicaksono, P. and Hafizt, M. (2017). Dark target effectiveness for dark-object subtraction atmospheric correction method on mangrove above-ground carbon stock mapping. *IET Image Processing*, *12*(4), 582-587.

Wicaksono, P. and Lazuardi, W. (2018). Assessment of PlanetScope images for benthic habitat and seagrass species mapping in a complex optically shallow water environment. *International journal of remote sensing*, *39*(17), 5739-5765.

Zimmerman, R., V. Hill and C. Gallegos. 2015. Predicting effects of ocean warming, acidification and water quality on Chesapeake region eelgrass. Limnology and Oceanography. ***60*: 1781-1804.**
